# Deciphering the transcriptional regulatory networks that control size, color, and oil content in *Brassica rapa* seeds

**DOI:** 10.1186/s13068-020-01728-6

**Published:** 2020-05-18

**Authors:** Yue Niu, Limin Wu, Yanhua Li, Hualei Huang, Mingchao Qian, Wei Sun, Hong Zhu, Yuanfang Xu, Yonghai Fan, Umer Mahmood, Benbo Xu, Kai Zhang, Cunmin Qu, Jiana Li, Kun Lu

**Affiliations:** 1 College of Agronomy and Biotechnology, Southwest University, Beibei, Chongqing, 400715 China; 2 InnoTech Alberta, Hwy 16A & 75 St., PO Bag 4000, Vegreville, AB Canada; 3 Saskatoon Research Centre, Agriculture and Agri-Food Canada, Saskatoon, SK Canada; 4 Institute of Characteristic Crop Research, Chongqing Academy of Agricultural Sciences, Chongqing, 402160 China; 5 College of Life Sciences, Yangtze University, Jingzhou, 434025 Hubei China; 6 Academy of Agricultural Sciences, Southwest University, Chongqing, 400715 China; 7 Engineering Research Center of South Upland Agriculture, Ministry of Education, Chongqing, 400715 China

**Keywords:** *Brassica rapa*, Oil content, Regulatory network, Seed color, Seed size

## Abstract

**Background:**

*Brassica rapa* is an important oilseed and vegetable crop species and is the A subgenome donor of two important oilseed *Brassica* crops, *Brassica napus* and *Brassica juncea*. Although seed size (SZ), seed color (SC), and oil content (OC) substantially affect seed yield and quality, the mechanisms regulating these traits in *Brassica* crops remain unclear.

**Results:**

We collected seeds from a pair of *B. rapa* accessions with significantly different SZ, SC, and OC at seven seed developmental stages (every 7 days from 7 to 49 days after pollination), and identified 28,954 differentially expressed genes (DEGs) from seven pairwise comparisons between accessions at each developmental stage. *K*-means clustering identified a group of cell cycle-related genes closely connected to variation in SZ of *B. rapa*. A weighted correlation analysis using the WGCNA package in R revealed two important co-expression modules comprising genes whose expression was positively correlated with SZ increase and negatively correlated with seed yellowness, respectively. Upregulated expression of cell cycle-related genes in one module was important for the G_2_/M cell cycle transition, and the transcription factor *Bra.A05TSO1* seemed to positively stimulate the expression of two *CYCB1;2* genes to promote seed development. In the second module, a conserved complex regulated by the transcription factor TT8 appear to determine SC through downregulation of *TT8* and its target genes *TT3*, *TT18*, and *ANR*. In the third module, WRI1 and FUS3 were conserved to increase the seed OC, and *Bra.A03GRF5* was revealed as a key transcription factor on lipid biosynthesis. Further, upregulation of genes involved in triacylglycerol biosynthesis and storage in the seed oil body may increase OC. We further validated the accuracy of the transcriptome data by quantitative real-time PCR of 15 DEGs. Finally, we used our results to construct detailed models to clarify the regulatory mechanisms underlying variations in SZ, SC, and OC in *B. rapa*.

**Conclusions:**

This study provides insight into the regulatory mechanisms underlying the variations of SZ, SC, and OC in plants based on transcriptome comparison. The findings hold great promise for improving seed yield, quality and OC through genetic engineering of critical genes in future molecular breeding.

## Background

Seeds are the most important harvested organ in many crop plants, storing large amounts of essential substances used by humans, and contain genetic materials that could be potentially used to improve crops. In angiosperms, seed development is initiated by a double fertilization event in which one of the two spermatids unites with an egg cell, while the other fertilizes the diploid central cell to form a triploid endosperm [1]. In monocotyledons and some dicotyledonous species, the endosperm occupies most of the mature seed, but in most dicotyledons, the embryo consumes a large amount of endosperm and occupies the bulk of the mature seed [2].

Seed size (SZ) is an important factor in crop yield, not only directly affecting the yield of seed crops, but also positively influencing seed germination and seedling growth in all species [2]. To date, several pathways regulating this significant agronomic trait have been identified and characterized. In the HAIKU (IKU) pathway, a classical SZ pathway controlling endosperm development, the triple mutant *iku1*/*iku2*/*mini3* of *Arabidopsis thaliana* (Arabidopsis) shows reduced SZ due to precocious cell formation of the endosperm [3, 4]. Furthermore, the MINI3 pathway directly regulates *CYTOKININ OXIDASE 2* (*CKX2*), inhibiting the activity of functional cytokinin (CK) to maintain endosperm growth [5]. SHORT HYPOCOTYL UNDER BLUE 1 (SHB1) is recruited by the promoters of *IKU2* and *MINI3* to promote their transcription. CKX2 is also regulated epigenetically [5, 6]. In the ubiquitin–proteasome pathway, the ubiquitin receptor DA1 acts synergistically with the E3 ubiquitin ligases DA2 or BIG BROTHER (BB)/ENHANCER OF DA1 (EOD1) to degrade various substrates and thereby reduce SZ by restricting cell proliferation [7]. In addition, DA1 functions synergistically with unknown E3 ubiquitin ligases to ubiquitinate UBIQUITIN-SPECIFIC PROTEASE 15 (UBP15), encoded by SUPPRESSOR OF DA1 (SOD2), resulting in small seeds and organs [6]. Overexpression of two transcription factors (TFs), TRANSPARENT TESTA GLABRA2 (TTG2), and KLU/CYP78A5, result in larger seeds due to positive regulation of cell expansion and cell proliferation in Arabidopsis [8]. In rice (*Oryza sativa*), the SQUAMOSA PROMOTER BINDING PROTEIN-LIKE (OsSPL13) TF promotes cell expansion and cell elongation to augment grain length and weight [9]. By contrast, APETALA2 (AP2) TFs reduce SZ through negative control of cell expansion [10]. In addition, plant hormones such as brassinosteroid (BR) and CK also play key roles in SZ variation. Overexpression of genes involved in BR biosynthesis leads to bigger seeds in Arabidopsis and rice, whereas mutants of BR biosynthesis genes show reduced SZ [11]. In Arabidopsis, the simultaneous silencing of the CK response factors *AHK2*, *AHK3*, and *CRE1* produces larger but fewer seeds [12]. In *Brassica* crops, however, although several pathways associated with SZ variation have been identified, the molecular mechanism underlying this trait, in terms of cell cycle and cell division pathways, remains largely unknown.

Yellow seed is a desirable trait of *Brassica* species due to the higher oil and protein content, but lower fiber content compared with dark seed [13]. Oxidized procyanidins are the key pigments in seed color (SC) formation in plants, and they are secondary products in the flavonoid and phenylpropane biosynthesis pathways [14]. Numerous *TRANSPARENT TESTA* (*TT*) genes, which participate in various stages of flavonoid synthesis, have been identified in Arabidopsis [15, 16]. A complex structure, composed of MYB, basic helix–loop–helix (bHLH) TFs, and WD40 proteins, has been identified as a crucial factor in determining SC by regulating the flavonoid biosynthesis pathway [17]. Although several homologous *TT* genes have been identified in *Brassica* crops, the regulatory mechanism of SC formation is still not particularly clear [18, 19], retarding the progress of yellow-seeded breeding of *Brassica* crops. In addition, *Brassica* species have been widely cultivated as oilseed crop, such as *Brassica napus*, *Brassica juncea*, and *Brassica rapa*. Triacylglycerols (TAG), as the main form of seed oil, not only play an important role in edible oil, but also can be used as a biofuel or biodiesel for industrial production [20]. Since 1961, when the TAG biosynthesis pathway was first proposed by Kennedy, the synthesis of glycerol has become a hallmark of lipid biochemistry [21]. In Arabidopsis, the TAG biosynthetic, metabolic, and degradation pathways have been studied comprehensively [22], and the genes encoding most of the enzymes involved in TAG metabolism have been and cloned characterized [23]. However, the molecular mechanism underlying fatty acid (FA) accumulation in seeds of *Brassica* crops remains poorly understood, resulting in a slow improvement of breeding for high oil content (OC) in *Brassica* crops.

*Brassica rapa* (AA, 2*n *= 20) is an important oil and vegetable *Brassica* crop that is widely cultivated worldwide [24]. It is also the diploid parental species of two important oilseed *Brassica* crops, *B. napus* (AACC, 2*n *= 38) and *B. juncea* (AABB, 2*n *= 36) [25]. Therefore, unravelling the regulatory mechanisms governing seed development and SZ in *B. rapa* would lay a solid foundation for breeding high-yield *Brassica* crops and related plants. Hence, we employed a pair of *B. rapa* accessions with significant differences in SZ, SC, and OC to analyze the transcriptomic variations of seeds at seven seed developmental stages. These results provide insight not only into the regulatory mechanism influencing SZ by regulating cell cycle progression, but also into the molecular mechanism of SC formation regulated by flavonoid biosynthesis, and that of OC difference caused by FA accumulation in *B. rapa*.

## Results

### Phenotypic variations between two *B. rapa* accessions

**Fig. 1:**
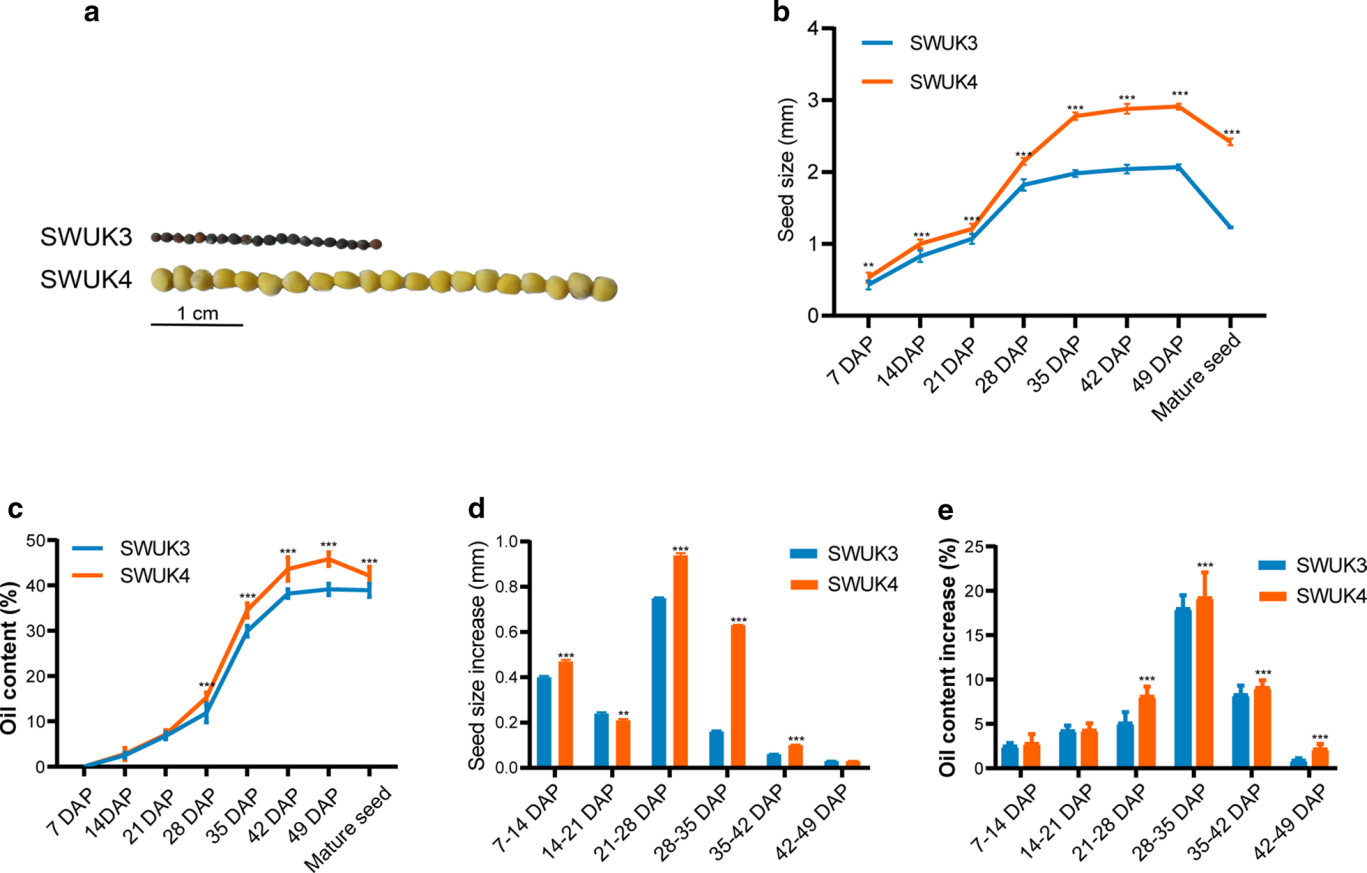
Phenotypic comparison between two *B. rapa* accessions. **a** Comparison of SZ and SC in SWUK3 and SWUK4. **b** Comparison of SZ of seeds sampled at seven seed developmental stages and dry mature seeds. **c** Comparison of OC in seeds sampled at seven seed developmental stages and dry mature seeds. **d** Comparison of SZ increase between two adjacent sampling stages. **e** Comparison of OC increase between two adjacent sampling stages. Values are the mean ± standard error (SE) of three biological replicates. Student’s *t*-test was used for statistical analysis of data from the two *B. rapa* accessions (**P < 0.01; ***P < 0.001)

The two *B. rapa* accessions were remarkably different in both SC, SZ and OC after harvest (Fig. 1a–c; Additional file 1: Table S1). The SC of *B. rapa* accession SWUK4 was yellow, whereas that of accession SWUK3 was black (Fig. 1a). The final SZ of SWUK4 seed (mean ± standard error of the longest dimension, 2.42 ± 0.05 mm) was significantly higher than that of SWUK3 (1.23 ± 0.01 mm), representing an approximately two-fold difference. And the fresh seeds of SWUK4 were visibly larger than those of SWUK3 at all seven sampling stages (Fig. 1b). Moreover, near-infrared reflection spectroscopy (NIRS) assays indicated that mature seed OC was significantly higher in SWUK4 (42.15 ± 1.97%) than in SWUK3 (38.93 ± 1.44%) (Fig. 1c). Gas chromatography (GC) measurements also identified differences in the abundances of the FA components (palmitic acid, stearic acid, oleic acid, linoleic acid, and linolenic acid) between the two accessions (Additional file 1: Table S1).

The speed of seed development is an important factor affecting the seed-filling process in *B. rapa*. The SZ of accession SWUK3 showed the greatest increase at 21–28 days after pollination (DAP) (0.75 ± 0.004 mm), followed by 7–14 DAP (0.4 ± 0.006 mm), whereas that of SWUK4 varied most at 21–28 DAP (0.94 ± 0.009 mm), followed by 28–35 DAP (0.63 ± 0.002 mm) (Fig. 1d), suggesting that the critical stage for SZ formation was 21–28 DAP in both *B. rapa* accessions, and the following stage, 28–35 DAP, was also quite important for the larger-seed accession. In addition, the two accessions showed the greatest increase in seed OC at 28–35 DAP (SWUK3: 18.02 ± 1.49%, SWUK4: 19.24 ± 2.84%) (Fig. 1e). The seed OC of SWUK4 increased greater than that of SWUK3 in all 7 developmental stages, especially 21–49 DAP (Fig. 1e). These results suggested that the critical stage for seed OC formation might be 28–35 DAP in *B. rapa*.

The silique length (SL) and thousand-seed weight (TSW) of the two *B. rapa* accessions were also significantly different. The SL of SWUK4 (40.90 ± 1.14 mm) was 1.6-fold longer than that of SWUK3 (25.00 ± 0.36 mm) (Additional file 1: Table S1), and the TSW of SWUK4 (7.06 ± 0.14 g) was approximate 2.9-fold greater than that of SWUK3 (2.42 ± 0.05 g) (Additional file 1: Table S1).

### Transcriptome sequencing analysis

To generate transcriptional networks and identify key regulatory genes regulating SZ, SC, and OC in *B. rapa*, we collected 42 seed samples from two distinct accessions at seven seed developmental stages. Transcriptome sequencing generated a total of 320 Gb of raw data, including 1076.28 million reads, averaging 7.62 G and 25.63 million reads per sample (Additional file 1: Table S2). Filtering of low-quality and contaminant reads left 1034 million clean reads, with an average of 24.64 million reads per sample. The G + C content of all samples varied from 44.39% to 51.51%, and the range of Q30 was from 91.12% to 94.28%. After mapping, three samples (one sampled from SWUK3 at 14 DAP, the rest sampled from SWUK4 at 14 and 21 DAP) were removed because they had unique mapping rates of < 70%. This resulted in an average unique mapping rate of 87.27% (Additional file 1: Table S2), indicating that the quality of our transcriptome sequencing reads was good enough for further regulatory network construction and identification of DEGs.

We eliminated one sample from SWUK4 at 49 DAP from further analysis because the correlation coefficient calculated for two of its biological replicates was only ~ 0.62, suggesting that this sample may have been contaminated. The remaining 38 samples all showed high reproducibility (*r*^2^ > 0.9) among biological replicates at each seed sampling stage (Additional file 2: Fig. S1). The correlations among the samples harvested at different seed developmental stages were much lower than those among biological replicates. Furthermore, the samples could be divided into two distinct groups in both *B. rapa* accessions—samples collected at 7, 14, and 21 DAP grouped together, whereas those from the other four time points formed a second group—suggesting that there might be a clear transition event at 21–28 DAP during seed development in *B. rapa* (Additional file 2: Fig. S1b,d).

### Identification of DEGs

**Fig. 2:**
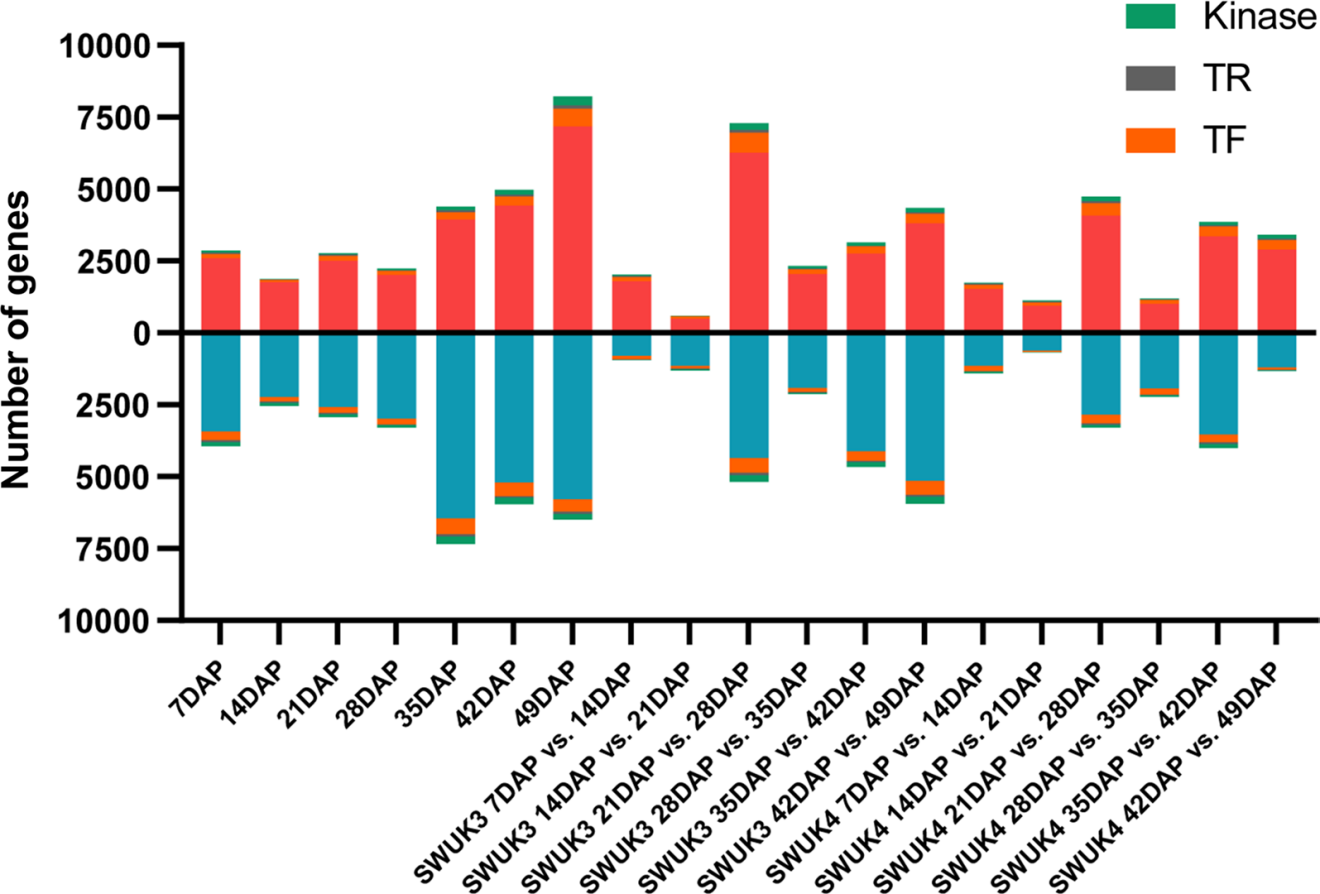
Number of DEGs. Classification of TF, TR, and kinase genes was performed using iTAK v1.6, (http://itak.feilab.net)

Through comparing the same seven seed developmental stages between the two accessions and the adjacent stages in each accession, a total of 28,954 unique DEGs were identified (Fig. 2, Additional file 1: Table S3). In the former of comparison, the number of DEGs rose over the course of seed development, suggesting that the variation in seed transcriptome between the two accessions gradually increased from seed formation to maturation (Fig. 2). The highest number of DEGs, 14,725, was observed at 49 DAP, and the number of downregulated DEGs was higher than that of upregulated DEGs at six stages, with the exception being 49 DAP (Fig. 2). In the later comparison, both two accessions had the highest number of DEGs at 21 DAP vs. 28 DAP (SWUK3: 12,468, SWUK4: 8040) (Fig. 2, Additional file 1: Table S3), and the least number of DEGs at 14 DAP vs. 21 DAP (SWUK3: 1906, SWUK4: 1797) (Fig. 2, Additional file 1: Table S3). These results suggested that the 21–28 DAP might be the most important seed developmental stage in *B. rapa*, duo to the greatest number of DEGs. According to the transcriptome comparison between samples at adjacent developmental stages, the change of DEG number showed similar trend in two *B. rapa* accessions (Fig. 2), suggesting that there was not obvious difference of the seed developmental stage between the two accessions.

An analysis of TFs, transcription repressors (TRs), and kinases using the online program iTAK identified a total of 3053 TFs, 602 TRs, and 1500 kinase genes in *B. rapa* (Additional file 1: Table S4). Among the DEGs, we detected 2261 unique differentially expressed TFs, indicating that the transcription of TFs (*P* = 2.48E−12) was regulated to a greater degree during seed development than was that of other genes, based on a Chi square test of the ratio between differentially expressed TFs and DEGs versus that between all TFs and all *B. rapa* genes. Among the TFs in *B. rapa*, the AP2/ERF–ERF, MYB, and bHLH families seem to play relatively important roles in seed development, as 169, 136, and 130 members of these families, respectively, were differentially expressed between the two accessions. Similar to TFs, the expression of 889 unique kinase genes and 355 unique TRs was also readily reprogrammed during seed development, but the significance levels (*P* = 6.33E−5 for kinase genes and *P* = 0.04 for TRs) determined using Chi square tests were lower than those for TFs.

### Gene Ontology (GO) enrichment analyses of DEGs

GO enrichment analysis revealed that the genes upregulated at 35, 42, and 49 DAP were significantly enriched in the term “fatty acid biosynthetic process” (GO:0006633). Given that OC differed significantly between the two *B. rapa* accessions, we deduce that transcriptional variation of these genes may play a crucial role in FA accumulation in *B. rapa*. Another group of upregulated genes may result in larger seeds through mechanisms involving the cell cycle regulation and CK signaling pathway, as they showed enrichment in “response to cytokinin stimulus” (GO:0009735), “cell cycle” (GO:0007049), “cell division” (GO:0051301), “M phase” (GO:0000279), “M phase of mitotic cell cycle” (GO:0000087), and “regulation of cyclin-dependent protein kinase activity” (GO:0000079) at the middle to late stages of seed development. Among the downregulated genes, important functions related to SC could be identified (Additional file 1: Table S5, S6). The GO enrichment analyses showed that “phenylpropanoid biosynthetic process” (GO:0009699) and “flavonoid biosynthesis process” (GO:0009813) were enriched at all stages of seed development according to both methods, except at 49 DAP in the GO enrichment analysis. These results indicated that a reduction of pigment biosynthesis and accumulation may be the cause for the yellow seeds of *B. rapa* accession SWUK4 (Additional file 1: Table S5).

GO enrichment analysis of DEGs obtained from comparison of the adjacent stages revealed that the up-regulation genes of 21 DAP vs. 28 DAP and 28 DAP vs. 35 DAP in both two accessions were significantly enriched in GO term “fatty acid biosynthetic process” (GO:0006636), “lipid biosynthetic process” (GO:0008610), and “cellular lipid metabolic process” (GO:0044255), suggesting that 21–28 DAP and 28–35 DAP may be the key stages of seed OC information in *B. rapa*. Genes down-regulated in 7 DAP vs. 14 DAP of SWUK3 and up-regulated in 21 DAP vs. 28 DAP of SWUK4 were significantly enriched in GO terms “cell cycle” (GO:0007049) and “cell division” (GO:0051301), implying that the key stages for SZ increase were different between the two *B. rapa* accessions. (Additional file 1: Table S6).

### Identification of expression patterns in DEGs by *K*-means clustering

**Fig. 3:**
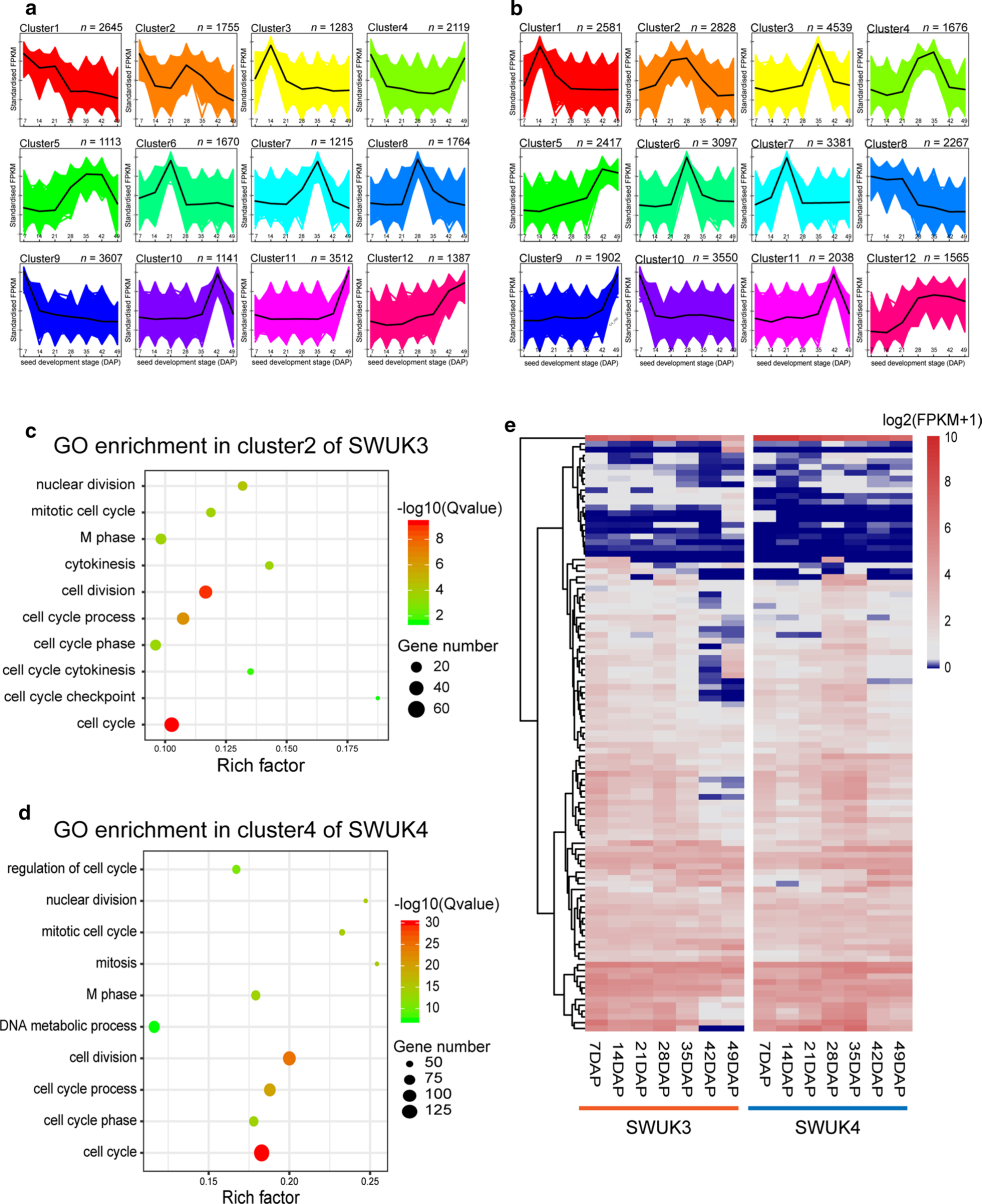
*K*-means clustering of DEGs. **a**, **b** Twelve clusters of **a** SWUK3 and **b** SWUK4 based on FPKM values at seven seed developmental stages. **c**, **d** GO enrichment analysis results for genes in **c** cluster 2 of SWUK3 and **d** cluster 4 of SWUK4. **e** Heatmap of expression of cell cycle core genes in *B. rapa*. Gene expression levels were transformed with the log_2_(FPKM + 1). All genes are listed in Additional file 1: Table S7

To identify DEGs with similar expression patterns during seed development, we performed two independent *K*-means clustering tests for 28,954 DEGs in two accessions (Fig. 3a, b), and generated 12 optimal clusters for each accession. We identified several clusters with similar expression patterns between the two accessions, such as cluster 5 of SWUK3 and cluster 12 of SWUK4, both of which showed gene transcription levels that increased continuously with seed development (Fig. 3a, b). GO and KEGG enrichment analyses revealed that these genes were enriched in lipid storage, seed oil body biogenesis, and related pathways, suggesting that the expression levels of genes related to lipid biosynthesis elevated since 21 DAP in SWUK3, and 14 DAP in SWUK4, and both peaked at 35 DAP, which was consistent with the GO results of DEGs obtained from comparison of the adjacent stages in each accession.

**Fig. 4:**
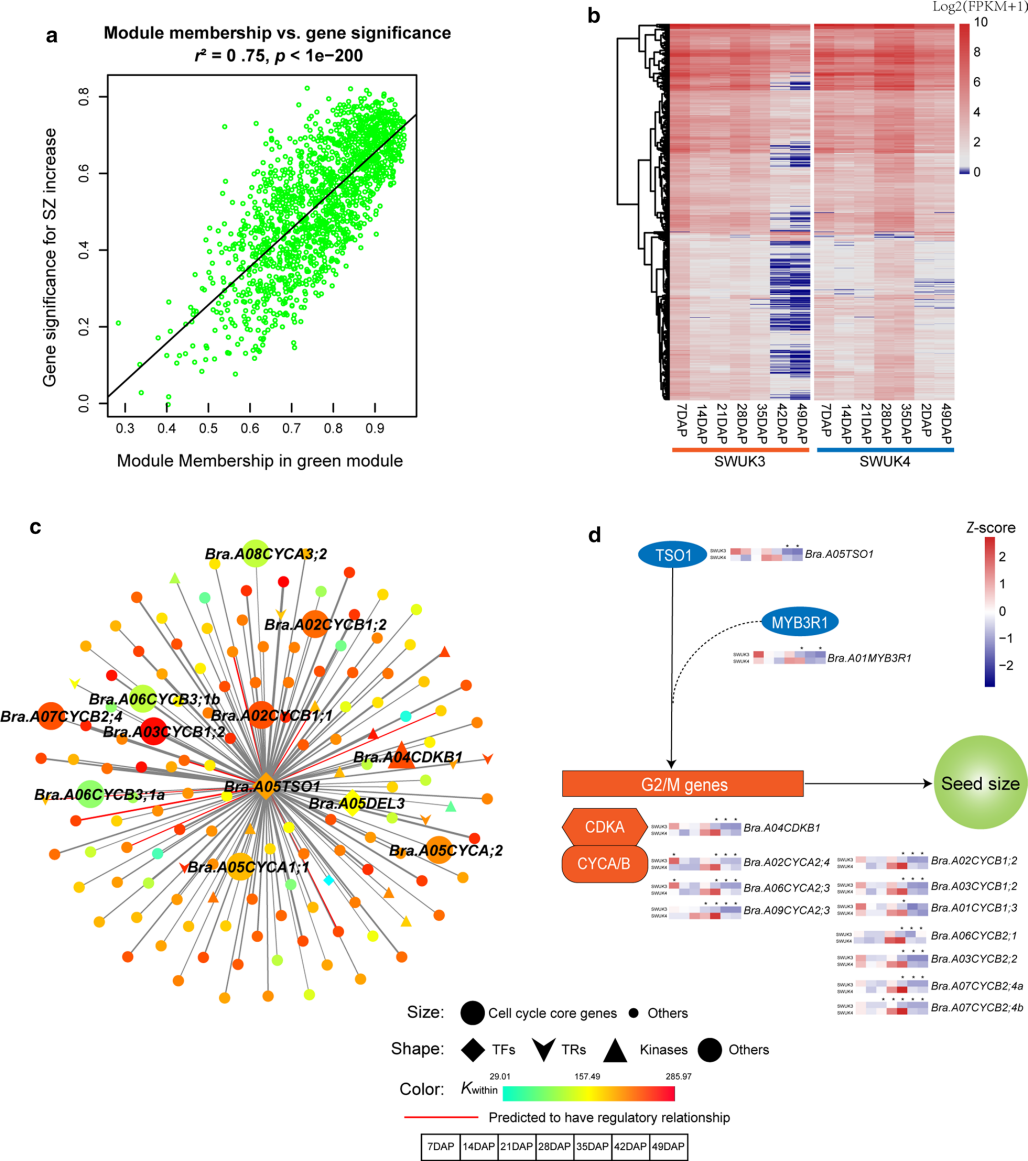
The WGCNA MEgreen module is significantly associated with SZ formation. **a** Scatter plot of the correlation of module membership (correlation coefficients between genes with MEgreen module) and gene significance (correlation coefficients between genes with trait of SZ increase). **b** Heatmap of MEgreen module genes in two *B. rapa* accessions, which was displayed based on log_2_(FPKM + 1). **c** Primary co-expression network of *Bra.A05TSO1*. Square, down arrow, triangle, and disc represent TF, TR, kinase, and other genes, respectively. The edge width represents the weight value between the two nodes: the higher the value of the weight between the nodes, the wider the edge. The regulatory relationships between the two nodes obtained from PlantRegMap are represented by red edges. **d** The regulatory model of TSO1 controlling seed size, with the heatmap of genes profiles in the two accessions. TSO1 play an important role in regulating the genes of G_2_/M phase, and MYB3R1 may bind to TSO1

A comparison of gene clusters in the two accessions revealed an interesting result in cluster 2 of SWUK3 and cluster 4 in SWUK4. Although the two clusters have different expression patterns, they were significantly enriched in similar functions involved in regulating the SZ, including cell cycle, cell division, and mitosis (Fig. 3c, d). In Arabidopsis, a total of 59 genes have been identified as core genes regulating the cell cycle and cell division. Based on a BLASTP analysis, we identified 101 homologs of these genes in *B. rapa* (Additional file 1: Table S7). Among these, the majority of positive regulatory genes showed similar patterns upon *K*-means clustering: they were highly transcribed at 7 and 28 DAP in SWUK3, but at 21–35 DAP in SWUK4 (Fig. 4e). Notably, the expression patterns were consistent with the respective phenotypic variations of SZ in the two accessions (Fig. 1d). Therefore, duration of cell cycle-specific gene expression at different stages may contribute to the difference of *B. rapa* seed SZ, and merit further investigation.

### Construction of co-expression networks

In crops, complex traits are generally regulated by several transcriptional networks. To identify the co-expression networks associated with our target traits, we used the R WGCNA software based on the fragments per kilobase per million mapped (FPKM) expression matrix and phenotypic data of six traits: developmental stage, SZ, SZ increase, seed yellowness, OC and OC increase. The sample clustering and correlation coefficients revealed strong repeatability among the biological replicates, and no outliers needed to be removed (Additional file 2: Fig. S2a). The results were also in agreement with those from the calculation of correlation coefficients, indicating that the seed samples from 7–21 DAP were grouped together, whereas the samples from the remaining stages formed a second group, again suggesting that seed transcriptome reprogramming is closely related to seed developmental stage, but weakly affected by accession.

In the WGCNA pipeline, pickSoftThreshold calculation revealed that the optimal soft threshold was 18, where the fitting curve approached 0.9 (Additional file 2: Fig. S2b). Then, we used the automatic blockwiseModules network construction approach to identify co-expression modules (Additional file 2: Fig. S2c). This allows the visualization of modules by color scheme, showing genes that are highly correlated in the same color, and genes that are weakly correlated in different colors (Additional file 2: Fig. S2d). The module construction process demonstrated that our functional color modules were clearly divided. After merging of modules with similar expression pattern, this process produced a total of 15 color modules, each composed of genes with similar expression patterns over time (Additional file 2: Fig. S3a).

As shown in Additional file 2: Fig. S3a, the abovementioned six traits (developmental stage, SZ, SZ increase, seed yellowness, OC and OC increase) showed significant correlation with different modules. After enrichment analysis of GO for each color module (Additional file 1: Table S8), the genes of the module defined as MEgreen, which had the highest correlation with SZ increase (*r*^2^ = 0.73, *P* = 2E−7), were significantly enriched in “cell cycle” (GO: 0007049), “cell division” (GO: 0051301), “regulation of cell cycle” (GO: 0051726), and “DNA replication” (GO: 006260) (Additional file 2: Fig. S3b). This indicated that the genes related to cell cycle participated in the positive regulation of seed growth rate. The functions of genes in the MEsalmon color module, which showed a significant negative correlation with seed coat transparency (*r*^2^ = –0.69, *P* = 1E−6), were enriched in the terms “flavonoid metabolic process” (GO: 0009812), “flavonoid biosynthetic process” (GO: 0009813), “phenylpropanoid metabolic process” (GO: 0009698), and “phenylpropanoid biosynthetic process” (GO: 0009699) (Additional file 2: Fig. S3c). This revealed that flavonoids negatively regulate yellow seed coat, such as that found in SWUK4. Furthermore, the MEbrown module, which is closely correlated with SZ (*r*^2^ = 0.7, *P* = 1E−6) and OC increase (*r*^2^ = 0.7, *P* = 1E−6), was enriched in “photosynthesis” (GO: 015979), “photosynthesis, light harvesting” (0009765), “lipid biosynthetic process” (GO: 0008610), and “fatty acid biosynthetic process” (GO: 0006633) (Additional file 2: Fig. S3d). This demonstrated that lipid synthesis occurs in the late stages of seed development in *B. rapa*, and requires photosynthesis to provide necessary materials and energy.

### Co-expression modules regulating SZ and SC

Co-expression network construction indicated that a SZ increase was most highly correlated with the MEgreen module (*r*^2^ = 0.73, *P *= 2E−7) (Additional file 2: Fig. S3a). The expression profiles of a large number of genes were highly correlated with both the module eigengene (average expression profile of module genes) and SZ in the MEgreen module (Fig. 4a). GO enrichment analysis indicated that the MEgreen module genes were significantly enriched in “cell cycle” (GO:0007049) and “cell division” (GO:0051301) (Additional file 2: Fig. S3b). A heatmap based on the expression levels of the MEgreen module genes revealed that the module eigengene of the MEgreen module was similar to the average expression profiles of cluster 3 in SWUK3 and cluster 4 in SWUK4 from the *K*-means clustering results (Figs. 3a, b, 4b), suggesting that the SZ of *B. rapa* was regulated mainly by genes related to the cell cycle and cell division.

To identify hub genes in our modules of interest, we first assessed gene connectivity (*K*_within_) on the basis of the absolute value of Pearson’s correlations. We then considered genes with the top 30% *K*_within_ in each module as hub genes of those modules. In the 800 genes of the MEgreen module, the *K*_within_ values ranged from 29.01 to 285.97. Based on the iTAK results, a total of 21 TFs, 17 TRs, and 36 kinase genes were identified in this module (Additional file 2: Fig. S4a). Two TF genes with high *K*_within_ values, *Bra.A05TSO1* (BraA05g024430, *K*_within_ = 217.73) and *Bra.A09GRAS* (BraA09g015380, *K*_within_ = 201.97), were selected as hub genes of the MEgreen module. Their Arabidopsis orthologs are *AtTSO1* (AT3G22780) and *AtSCL28* (AT1G63100, a TF of the GRAS family), respectively. In the primary *Bra.A05TSO1* network, *Bra.A05TSO1* was directly co-expressed with 147 genes, including 2 TFs (belonging to the E2F and B3 families, respectively), 7 TRs, and 11 kinase genes (Fig. 4c). The primary network of *Bra.A09GRAS* contained 248 co-expressed genes, including 5 TFs (2 B3, 1 GARP and 1 TUB family), 9 TRs, and 15 kinase genes (Additional file 2: Fig. S4b).

**Fig. 5:**
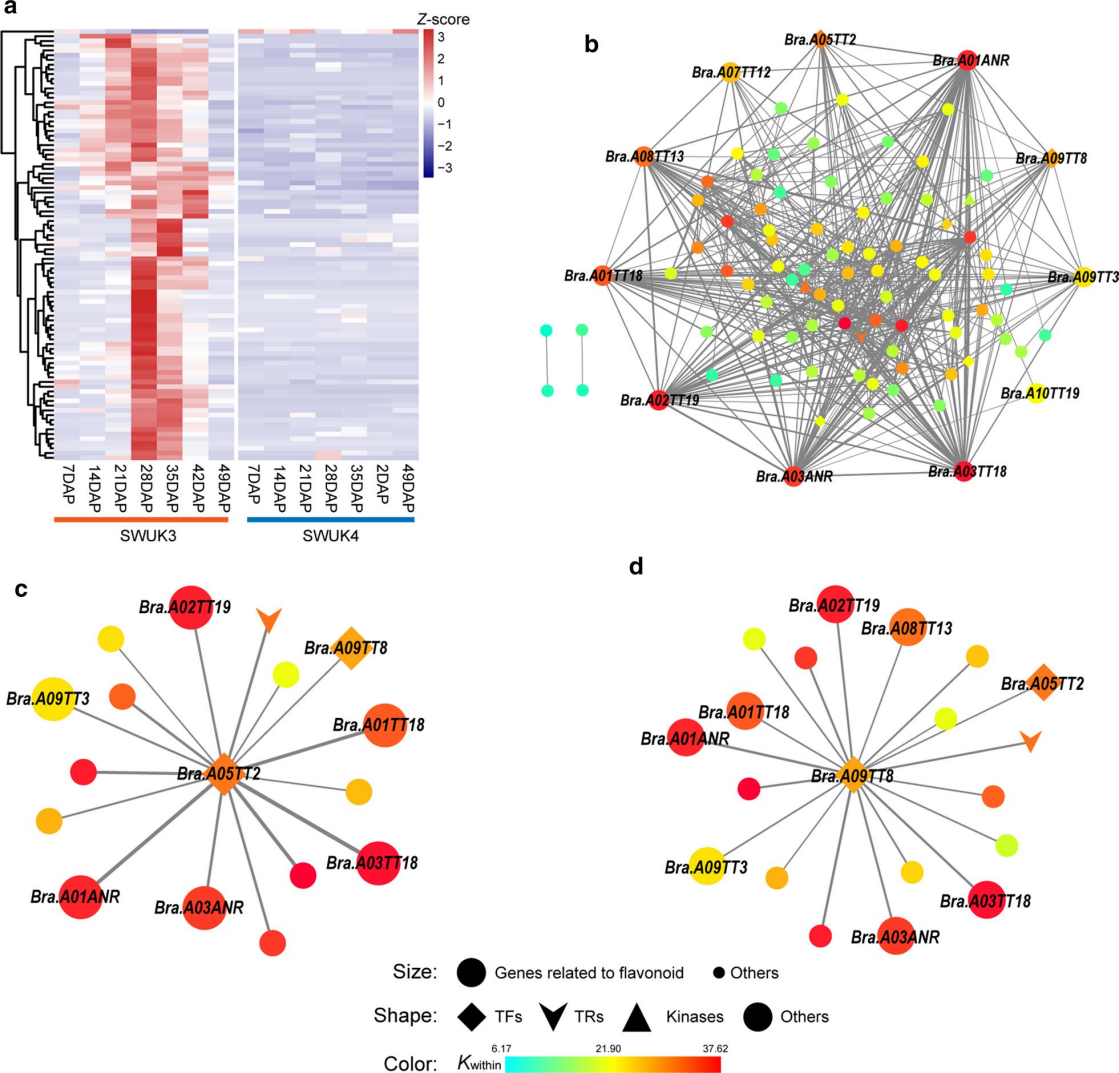
The WGCNA MEsalmon module is negatively related to SC formation. **a** Heatmap of MEsalmon module genes in two *B. rapa* accessions. Gene expression levels are normalized by *Z*-score. **b** The MEsalmon co-expression network. **c** Primary co-expression network of *Bra.A05TT2.***d** Primary co-expression network of *Bra.A09TT8*. Square, down arrow, triangle, and disc represent TF, TR, kinase, and other genes, respectively. The edge width represents the weight value between the two nodes: the higher the value of the weight between the nodes, the wider the edge

Another module of interest was MEsalmon, which showed a significant negative correlation with seed yellowness (*r*^2^ = –0.69, *P* = 1E−6) (Additional file 2: Fig. S3a). The genes in this module were enriched in the GO terms “flavonoid biosynthetic process” (GO:0009813), “regulation of flavonoid biosynthetic process” (GO:0009962), and “phenylpropanoid biosynthetic process” (GO:0009699) (Additional file 2: Fig. S3c). The gene expression levels in this module were clearly lower in SWUK4 than in SWUK3 at most of the sampling stages (Fig. 5a). These results suggest that the formation of yellow seeds in SWUK4 is most likely due to weak expression or silencing of flavonoid biosynthesis pathway genes. The co-expression network of the MEsalmon module comprised 91 genes, including 5 TFs, 1 TR, and 2 kinase genes (Fig. 5b). The *K*_within_ values of these genes varied from 6.17 to 37.62. Two well-known phenylpropanoid biosynthetic pathway TF genes, *Bra.A05TT2* (BraA05g008220, R2R3-MYB, *K*_within_ = 30.23) and *Bra.A09TT8* (BraA09g028560, bHLH, *K*_within_ = 27.54), were identified as hub genes underlying SC formation (Fig. 5c, d).

MEbrown module had the highest correlation coefficient with OC increase (*r*^2^ = 0.7, *P* = 1E−6) (Additional file 2: Fig. S3a). Genes in this module were enriched in the GO terms “lipid biosynthetic process” (GO:0008610), “fatty acid biosynthetic process” (GO:0006633), and “lipid metabolic process” (GO:0006629) (Additional file 2: Fig. S3d). Most of the genes in this module were most expressed at 28 and 35 DAP in the two accessions, but they had higher expression level in late seed development of SWUK4 (high OC) than SWUK3 (low OC) (Additional file 2: Fig. S5a). It indicates that the critical period for seed oil synthesis in *B. rapa* is 28–35 DAP. The co-expression network of the MEbrown module comprised 2268 genes, including 209 TFs, 21 TRs, and 79 kinase genes. The *K*_within_ values of them from 6.33 to 511.45. Three TFs with the highest *K*_within_ values, *Bra.A03GRF5* (*BraA03g036220*, *K*_within_ = 433.30), *Bra.A09WRI1* (*BraA09g045300*, *K*_within_ = 432.83), and *Bra.A06FUS3* (*BraA06g038070*, *K*_within_ = 414.13), were selected as hub genes of the MEbrown (Additional file 2: Fig. S5b–d).

### Role of flavonoid pathway genes in SC formation

Because the abovementioned analysis suggested that the differences in SC and OC between the two *B. rapa* accessions might be caused by flavonoid pathway and FA biosynthesis genes, we performed a further expression comparison analysis of these two types of genes. All expression values were subjected to log_2_(FPKM + 1) transformation and *Z*-score normalization.

**Fig. 6:**
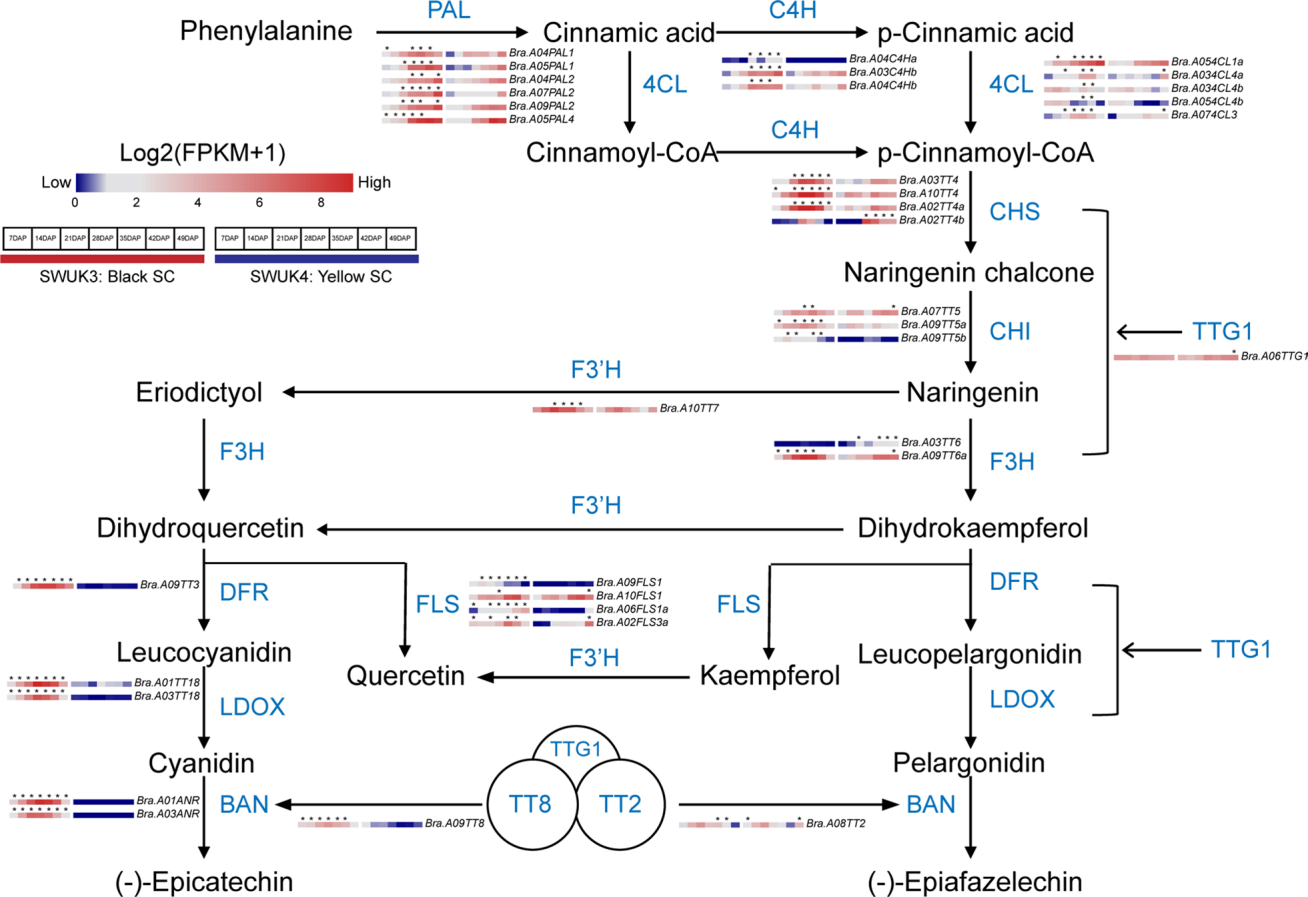
Flavonoid pathways, with enzymes encoded by genes involved in SC formation. Gene expression values in the heatmap were normalized by log_2_(FPKM + 1). All of these genes are listed in Additional file 1: Table S9. Asterisks (*) above the heatmap images indicate that a given gene is significantly more highly expressed in the *B. rapa* accession marked with an asterisk. Protein abbreviations: *PAL* phenylalanine ammonia lyase; *C4H* cinnamate 4-hydroxylase, *4CL* 4-coumarate coenzyme A ligase; CHS; chalcone synthase, *CHI* chalcone isomerase, *F3H* flavonol 3-hydroxylase, *F3′H* flavonol 3′-hydroxylase, *FLS* flavonol synthase, *DFR* dihydroflavonol-4-reductase, *LDOX* leucoanthocyanidin dioxygenase; *BAN* BANYULS, *TTG1* TRANSPARENT TESTA GLABRA1

For pathways involved in the formation of compounds contributing to SC (Fig. 6), there were 43 homologous genes in *B. rapa* (Additional file 1: Table S9). At the beginning of the pathway, the generation of *p*-cinnamoyl-CoA requires the three enzymes PAL, CH4, and 4CL, encoded by 14 genes in *B. rapa* (Additional file 1: Table S9). All of these genes showed significantly differential expression between the two accessions at one or more stages of seed development. Their expression patterns were similar, with expression increasing over time and being higher in the black-seeded accession SWUK3 at almost all stages. In the first step of flavonoid biosynthesis, the formation of *p*-cinnamoyl-CoA is catalyzed by CHS to produce the chalcone compound naringenin, which is then converted to its isomer naringenin in a reaction catalyzed by CHI. Next, F3H and F3′H catalyze reactions converting naringenin to dihydrokaempferol and then eriodictyol. Of the 12 genes encoding these four enzymes in *B. rapa*, only two (one *CHI* and one *F3H* gene) were not differentially expressed between the two accessions.

The downstream portion of flavonoid biosynthesis is divided into two branches. In one branch, dihydrokaempferol and eriodictyol are catalyzed by FLS to generate dihydrokaempferol and dihydroquercetin. Of the five genes encoding this enzyme, only one gene was not differentially transcribed between accessions. In the other branch of the pathway, dihydrokaempferol and dihydroquercetin are catalyzed to become (–)-epiafzelechin and (–)-epicatechin by the enzymes DFR, LDOX, and BAN, which are encoded by five *B. rapa* genes. Notably, four of these genes were silenced and the fifth showed dramatically decreased transcription in the yellow-seeded accession SWUK4 at all seed developmental stages, indicating that the mutation of epicatechin branch genes may block pigment accumulation in *B. rapa* seeds.

### Role of genes involved in lipid biosynthesis and storage in OC increase

**Fig. 7:**
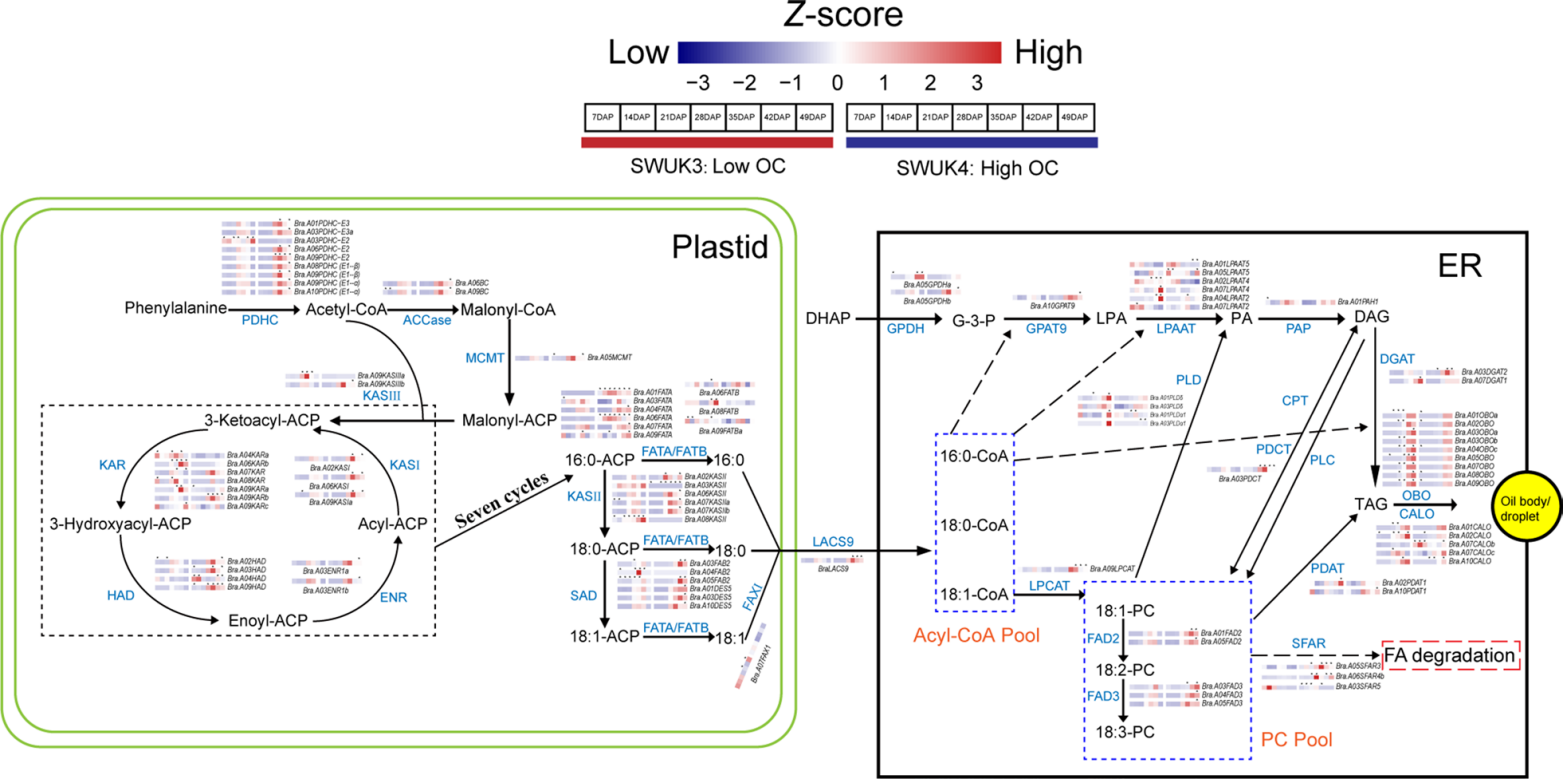
FA metabolism pathway genes regulating the OC. All the genes are listed in Additional file 1: Table S10. Gene expression values were normalized by *Z*-score. Asterisks (*) above the heatmap indicate that a given gene is expressed at significantly higher levels in the *B. rapa* accession marked with an asterisk. Protein abbreviations: *PDHC* pyruvate dehydrogenase complex, *ACCase* acetyl-CoA carboxylase, *MCMT* malonyl-CoA:ACP malonyltransferase, *ACP* acyl carrier protein, *KAS II* 3-ketoacyl-ACP synthase II, *KAR* ketoacyl-ACP reductase, *HAD* hydroxyacyl-ACP dehydrase, *ENR* enoyl-ACP reductase, *SAD* stearoyl-acyl carrier protein desaturase, *FATA/B* fatty acyl-ACP thioesterase A/B, *FAX1* plastid fatty acid export 1, *LACS9* long-chain acyl-CoA synthetase 9, *GPDH* glycerol-3-phosphate dehydrogenase, *GPAT9* glycerol-3-phosphate acyltransferase 9, *LPAAT* lysophosphatidic acid acyltransferase, *PAP* phosphatidic acid phosphatase, *DGAT* diacylglycerol acyltransferase; *LPCAT* lysophosphatidylcholine acyltransferase, *FAD2/3* fatty acid desaturase 2/3, *PLD* phospholipase D, *PDAT* phospholipid: diacylglycerol acyltransferase, *PDCT* phosphatidylcholine: diacylglycerol cholinephosphotransferase, *CPT* CDP-choline: diacylglycerol cholinephosphotransferase, *PLC* phospholipase C, *OBO* oil body oleosin, *CALO* caleosin, *SFAR* seed fatty acid reducer

In plants, lipid biosynthesis and accumulation in seeds can be divided into four steps (Fig. 7). (1) Pyruvate and other substances form C16-C18 FAs in the plastid. (2) FAs are transported into the cytoplasm, where they undergo elongation and desaturation of their carbon chains. (3) Various FAs and glycerol are catalyzed to synthesize triacylglycerols (TAG) and store them in the seed oil body, after which some are (4) degraded by products of the GDSL-type *Seed Fatty Acid Reducer* (*SFAR*) genes.

In the first step, genes shared similar expression patterns in both accessions, being more highly expressed at 28–49 DAP than at 7–21 DAP (Fig. 7). However, most genes encoding proteins involved in this step were generally more highly expressed in the higher-OC accession SWUK4 at each developmental stage, and could be found in cluster 12 of SWUK3 and cluster 5 of SWUK4 in *K*-means clustering, indicating that oil synthesis in *B. rapa* seed is mainly initiated at a later stage of seed development. In the second step, the four genes encoding the relevant catalytic enzymes [3-Ketoacyl-ACP Synthase II (KAS II), Stearoyl acyl Carrier Protein Desaturase (SAD), Fatty Acid Export 1 (FAX1), and Long Chain Acyl-CoA Synthetase 9 (LACS9)] were more highly expressed in SWUK4 (Fig. 7), suggesting that SWUK4 seeds have a larger acyl-CoA pool than SWUK3 seeds. Differential expression of genes encoding Lysophosphatidylcholine Acyltransferase (LPCAT) and FA Desaturase 2/3 (FAD2/3) between the two accessions may cause the significant variation in FA components, including oleic acid, linoleic acid, and linolenic acid, in mature seeds detected by GC analysis (Additional file 1: Table S1). In the third step, TAG is formed in the endoplasmic reticulum catalyzed by four enzymes, glycerol-3-phosphate acyltransferase 9 (GPAT9), 1-acylglycerol-3-phosphate acyltransferase (LPAAT), phosphatidic acid phosphatase (PAP), and diacylglycerol acyltransferase (DGAT) [26]. The majority of genes encoding these four enzymes were more highly expressed in SWUK4 than in SWUK3 at stages 28–49 DAP, implying that greater TAG biosynthesis and storage in seed oil body might be the cause for the higher OC in SWUK4. In the fourth step, SFAR genes negatively regulate FA storage and seed oil body size, leading to FA degradation. *B. rapa* has 12 *SFAR* genes, but only two of them showed significant upregulation in the higher-OC accession SWUK4 at late sampling stages, suggesting that this accession does not adopt the strategy of negatively regulating SFAR activity to increase OC. In addition, the overexpression of *SFAR* genes resulted in the increase of C18:1 and C20:1, and the decrease  of C18:2 and C18:3 proportions [27], which explained why the SWUK4 has the higher content of oleic acid and lower content of linoleic acid and linolenic acid in its mature seeds (Additional file 1: Table S1). However, whether the OC of accession SWUK4 could be increased by reducing FA degradation remains to be established.

### qRT-PCR validation

**Fig. 8:**
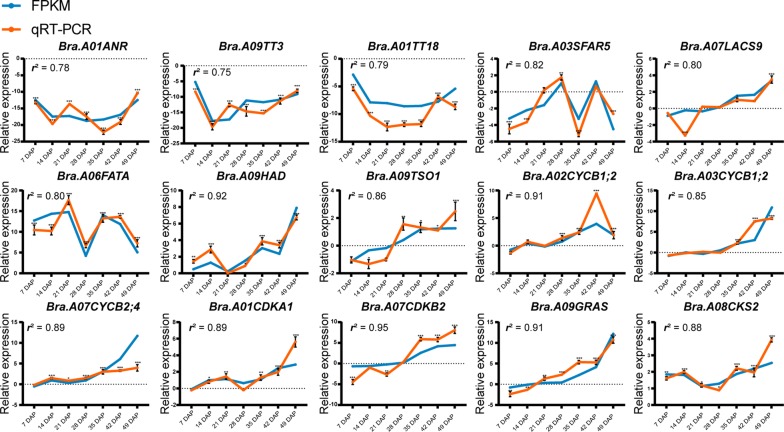
qRT-PCR validation of DEGs. Fifteen genes were selected for validation, including three encoding proteins involved in SC formation (*Bra.A01ANR*, *Bra.A09TT3*, and *Bra.A01TT18*) and four related to lipid biosynthesis and degradation (*Bra.A03SFAR5*, *Bra.A07LACS9*, *Bra.A06FATA*, and *Bra.A09HAD*); the remaining genes are related to the cell cycle. The blue lines represent RNA-seq results and the orange lines represent qRT-PCR results. Student’s *t*-test was used for statistical analysis of data from the two *B. rapa* accessions (*P < 0.05; **P < 0.01; ***P < 0.001)

The relative expression levels of the 15 key DEGs at the seven seed developmental stages were analyzed by qRT-PCR to assess the accuracy of the RNA-seq results. The relative expression patterns of DEGs tested were positively correlated with the fold change variations obtained from the RNA-seq results (Fig. 8). The correlation coefficients between qRT-PCR and RNA-seq for the 15 DEGs ranged from 0.75 to 0.92. Further comparison showed that all the lower correlation coefficients between the two approaches belonged to three flavonoid pathway genes, *Bra.A01TT18*, *Bra.A01ANR*, and *Bra.A09TT3*. Due to the silencing of the three genes in the yellow-seeded *B. rapa* accession, their FPKM values were zero, but had to be set to 0.001 for fold change calculation, leading to lower correlation coefficients. However, the remaining 12 genes associated with FA metabolism and cell cycle progression all showed better correlation than the flavonoid pathway genes, demonstrating the reliability and accuracy of our RNA-seq results.

## Discussion

### Cell cycle-related genes may regulate SZ in *B. rapa*

*B. rapa* is a model plant for *Brassica* species. Understanding the molecular mechanism regulating *B. rapa* SZ can provide essential reference information for other *Brassica* crops. In this study, we investigated these mechanisms in two *B. rapa* accessions with significant differences in seed development progression and final SZ. WGCNA co-expression network analysis showed that one expression module, i.e., the MEgreen module, had the highest correlation with SZ increase (Additional file 2: Fig. S3a), and the genes in the module were significantly enriched in cell cycle-related functions. Using the *K*-means clustering method, we obtained two clusters with different expression profiles but similar functions in the cell cycle from two *B. rapa* accessions. A comparison between the WGCNA and *K*-means clustering results indicated that both methods identified as critical genes those associated with the cell cycle, cell division, and mitotic cell cycle, and that these were significantly correlated with traits of interest, suggesting that transcriptional regulators of cell cycle-related genes might be potential targets for improvement of seed yield in *B. rapa* and related *Brassica* crops.

Cell cycle regulation plays a crucial role in the growth and development of plant organs, affecting both cell size and cell number. We identified 101 cell cycle core genes in *B. rapa*, most of which were more highly expressed in the large-seeded accession SWUK4 than in the small-seeded accession SWUK3 (Fig. 3e). The cyclin-dependent kinases (CDKs) play a central role in cell cycle regulation, and their activity is also controlled by regulatory subunits, such as cyclins and E2F TFs [28].

Previous studies have shown that SZ is regulated by cell cycle-related genes. A genome-wide association study of Arabidopsis SZ variation identified a B1-type CDK gene, *AtCYCB1;4*, and revealed that *AtCYCB1;4* overexpression plants produced larger seeds than wild-type plants [29]. Arabidopsis *AtCYCB1;2* is responsible for stimulating the G_2_/M transition and is critical for cell division cycle progression during seed development [30, 31]. *Brassica rapa* has four *CYCB1* genes (one *CYCB1;1*, two *CYCB1;2*, and one *CYCB1;3* genes), of which three, *Bra.A02CYCB1;2*, *Bra.A03CYCB1;2*, and *Bra.A01CYCB1;3*, showed higher expression levels in the large-seeded accession SWUK4 at seed developmental stages from 28 to 49 DAP, the fast-growing stages of seeds in *B. rapa*.

In addition, the expression patterns of the two *CYCB1;2* genes were positively correlated with SZ increase, suggesting that *CYCB1;2* might be a key regulatory target for SZ trait improvement in *Brassica* crops. Eight other *CDK* genes (including four *CYCB*, three *CYCA*, and one *CDKB* genes) were also differentially expressed between the two *B. rapa* accessions. Although they were not included in the co-expression networks, the relationship of their expression to that of genes in these networks is also worthy of further investigation given their importance in cell cycle regulation.

### The conserved DREAM complex may control seed size as an upstream coordinator

In the past 20 years, an evolutionarily conserved complex known as the DREAM complex has been identified as a master coordinator of cell cycle-dependent gene expression in animals [32]. DREAM consists of a core complex of five members, LIN9, LIN37, LIN52, LIN 53 (RBBP4), and LIN54, called MuvB (multivulva class B proteins) or MIP (MYB-interacting proteins) in *Caenorhabditis elegans* and mammals [32]. Perturbations in DREAM complex regulation trigger a shift of the balance from quiescence towards proliferation and result in increased mitotic gene expression that is frequently observed in human cancers.

The conserved function of the DREAM complex has also been identified in plants. In Arabidopsis, the homolog of *LIN54*, *AtTSO1*, coordinates with MYB3R1 to activate or inhibit cell cycle-related genes, and regulates the development of the root apical meristem and shoot apical meristem [33]. LIN54 is a core subunit of the DREAM complex. Downregulating *LIN54* expression disrupts the DREAM complex, thereby reducing the expression of G_2_/M cell cycle genes [34].

In this study, we constructed a co-expression network with the hub TF *Bra.A05TSO1*, and identified the regulatory relationship between *Bra.A05TSO1* and its 23 downstream target genes using PlantRegMap (Fig. 4c). *Bra.A05TSO1* could bind *cis*-regulatory elements in the promoters of two cell cycle core genes, *Bra.A02CYCB1;2* and *Bra.A03CYCB1;2*, to stimulate their expression (Fig. 4c), suggesting that the regulatory module TSO1-CYCB1;2 may contribute to the G_2_/M transition so as to control the cell cycle progression and affect the final SZ in plants, reminiscent of the similar mechanism adopted by LIN54 in animals (Fig. 4d). Hence, it is likely that the function of the DREAM complex in cell cycle regulation is conserved between plants and animals.

MYB binding with MuvB is a key factor in DREAM complex regulation of the cell cycle. When the cell leaves the S phase and enters the G_2_ phase, MYB binds the MuvB complex to activate genes required for mitosis [32]. However, B-MYB may also cause transcriptional suppression in mammals [30]. The Arabidopsis homologs of the Myb oncoprotein are the MYB3R (R1R2R3-MYB) TFs, which contain similar domains and also regulate the expression of G_2_ and M phase cell cycle genes [31]. Among the five Arabidopsis MYB3Rs, MYB3R1 not only can be redundant with MYB3R4 in transactivating the expression of specific genes at the G_2_ and M phases, including that of *CYCB1* [28, 35], but also can act as a repressor that inhibits the expression of cell cycle-related genes, similarly to MYB3R3 and MYB3R5 [32].

In our study, we identified 11 members of the *MYB3R* gene family. Though we did not identify *MYB3R* genes in the MEgreen module, we did identify *Bra.A05TSO1*, *Bra.A02CYCB1;2*, and *Bra.A01MYB3R1* (BraA01g005340) in cluster 6 of SWUK4 in the *K*-means clustering analysis, suggesting that the three genes have similar expression patterns in the larger-seeded accession. Hence, we propose a model of SZ regulation in *B. rapa* whereby the TF *Bra.A05TSO1* positively regulates *BraCYCB1;2* either directly or in coordination with *Bra.A01MYB3R1* (as in the DREAM complex in animals), and then promotes cell division to produce larger seeds (Fig. 4d).

### TT8 might be a conserved key target for creating yellow-seeded *Brassica* crops

Yellow seed color is a desirable trait with great potential for improving oil and protein content and meal quality in *Brassica* crops. Though several yellow-seeded varieties of *B. rapa*, *B. napus*, *B. juncea*, and *Brassica carinata* have been bred, and the inheritance of SC traits in these varieties has been studied, the molecular mechanism controlling the target trait remains to be unraveled. However, a similar trait regulated by the flavonoid pathway has been well characterized at the molecular level by utilizing *transparent testa* (*tt*) mutants, which carry mutations that hinder flavonoid accumulation and modify pigmentation in the Arabidopsis seed coat [36–38].

Our WGCNA identified a module, denoted MEsalmon, that is significantly negatively correlated with *B. rapa* SC (Additional file 2: Fig. S3a). The two TF genes *Bra.A09TT8* and *Bra.A08TT2* were identified as hub genes in this co-expression network (Fig. 5c, d). Previous studies had revealed that three TF regulatory genes, *AtTT2*, *AtTT8*, and *TTG1* (encoding a WD40 protein), could form an MYB-bHLH-WD40 (MBW) complex that activates proanthocyanidin (PA)-specific genes (such as *TT3*, *TT18*, and *ANR*) during seed coat development [39]. In our results, two of the genes encoding MBW complex members, *Bra.A08TT2* and *Bra.A06TTG1*, seem to be unimportant for SC formation in *B. rapa*, since their expression was either unregulated or higher in the yellow-seeded accession at one stage only. However, the product of the third MBW gene, *Bra.A09TT8*, may play a critical regulatory role in SC formation as a core TF in the flavonoid pathway. Our results indicated that *Bra.A09TT8* expression in the yellow-seeded accession was very weak, and significantly lower than in the black-seeded accession, at all seed developmental stages except at 49 DAP. Therefore, it is likely that the accumulation of the PA precursor epicatechin might be blocked due to the silencing of *Bra.A09TT8*, consistent with a previous study showing that the insertion of a transposable element in *BraTT8* caused a loss of function of BraTT8 in the yellow-seeded *B. rapa* var. yellow sarson [40]. Recent studies have also revealed that simultaneous natural mutations of two homologous *TT8* genes in allotetraploid *B. juncea* resulted in the yellow-seeded trait [41], and a similar mutant also could be created in *B. napus* using CRISPR/Cas9 technology [42].

We also observed that the regulatory network of *TT8* was well conserved in Arabidopsis and *Brassica* crops. In Arabidopsis seeds, the MBW complex AtTT8-AtTT2-AtTTG1 positively regulates PA synthesis by directly binding to the *cis*-regulatory elements of DFR, LODX, and BAN in the flavonoid biosynthesis pathway [43]. In the primary co-expression network of *Bra.A09TT8*, we identified three downstream target genes, *Bra.A09TT3* (BraA09g019440), *Bra.A01ANR* (*BraA01g029500*), and *Bra.A03ANR* (BraA03g064730), that were almost silenced in the yellow-seeded *B. rapa* accession during seed development. Though two *TT18* genes (*Bra.A01TT18* and *Bra.A03TT18*) in *B. rapa* were not involved in the *Bra.TT8*-containing primary network, a positive regulatory relationship between *TT8* and *TT18* could be observed due to their similar weak expression in the yellow-seeded *B. rapa* accession. A similar network has also been identified in *B. napus* [42, 44, 45], Thus, we infer that the regulatory network of the TT8-involved complex was evolutionarily conserved between Arabidopsis and *Brassica* crop species. Considering the divergence of the two genera, the conserved *TT8* genes might be optimal target loci for manipulation in the breeding of yellow-seeded varieties of *Brassica* and related crops.

### *GRF5*, *WRI1*, and *FUS3* may be key TF genes on increasing seed OC in *B. rapa*

*B. rapa* is one of the most important oil crop in the world. It is essential to explore the molecular mechanism on seed OC formation in *B.rapa*. Our WGCNA identified the MEbrown module, which was the highest correlation with OC increase and significantly associated with fatty acid biosynthsis (Additional file 2: Fig. S3a, d). Three TF genes *Bra.A03GRF5*, *Bra.A09WRI1*, and *Bra.A06FUS3* were identified as hub genes in this regulatory network (Additional file 2: Fig. S5b–d).

The hub TF *Bra.A03GRF5*, as the highest *K*_within_ value of TFs in MEbrown module, may be a novel target in the regulation of FA biosynthesis. In previous researches, GRFs were identified as transcription activators [46], and invoved in ovule formation and female reproductive development in *Arabidopsis* [47]. They were also expressed in rice embryo and maize ear [48, 49], suggesting that GRFs may play role in seed development regulation. In addition, GRF2-like genes were highly expressed in high seed OC accessions in *B. napus*, and overexpression of *BnGRF2* in *Arabidopsis* produced higher seed OC than wild type [50]. In our study, *Bra.A03GRF5* co-expressed directly with 36 genes related to TAG biosynthesis, such as *Bra.A07LACS9*, *Bra.A09PDHC (E1*-*β)* and *Bra.A02KASI*, which may be the downstream target genes of *Bra.A03GRF5*. (Additional file 2: Fig. S5b). Hence, it could be expected that *Bra.A03GRF5* may be involved in regulation of seed lipid biosynthesis, similar with *BnGRF2*.

In addition, *Bra.A09WRI1* and *Bra.A06FUS3* were also identified as hub TFs in MEbrown module, and co-expressed with genes involved in lipid biosynthesis (Additional file 2: Fig. S5c,d). Overexpression of *JcWRI1* increased *Jatropha* seed OC and seed mass [51]. Heterologous overexpression of homologs of *Arabidopsis AtWRI1*, such as *B. napus*, *Zea mays* and *Elaeis guineensis*, led to seed OC increase in *Arabidopsis* [52–54]. FUS3 is an well-known TF on enhancing TAG biosynthesis. The *fus3* mutant plants shown a decrease in lipid accumulation in *Arabidopsis* [55], and suppression of *BnFUC3* also decreased OC in *B. napus* [56]. Besides, previous study also shown that LEC1 was upstream of WRI1 and FUS3 to promote lipid synthesis [57, 58], and the WRI1 was also activated by FUS3 [59, 60]. In our study, *Bra.A07LEC1* was simultaneously occurred in the primary networks of *Bra.A09WRI1*, *Bra.A06FUS3* and *Bra.A03GRF5*. And the three TF genes were also directly co-expressed each other. Our results suggested the conservative function of WRI1 and FUS3 in *B. rapa*, and confirmed the reliability of the construction of our WGCNA results. However, it is interested and worth to explore that the role of GRF5 in seed OC increase and the interaction between the three TF genes.

## Conclusion

In this study, we identified 28,954 DEGs from transcriptome comparisons of seeds sampled from a pair of *B. rapa* accessions with different SZ, SC, and OC at seven seed developmental stages. Both *K*-means clustering and WGCNA identified a group of cell cycle-related genes whose expression was also positively correlated with SZ increase, and indicated that the TF *Bra.A05TSO1* may positively stimulate the expression of two *CYCB1;2* genes to increase the SZ through regulation of the G_2_/M transition. In a module whose expression was negatively correlated with seed yellowness, a conserved TT8-involved complex may determine the SC through downregulation of the key TF gene *TT8* and its targets *TT3*, *TT18*, and *ANR* in the flavonoid pathway. Moreover, in a module that is enriched in fatty acid biosynthesis, we identified a novel key TF *Bra.A03GRF5* may increase the seed OC, and found two conservative TFs *WRI1* and *FUS3* on regulating seed OC. Upregulated genes involved in triacylglycerol biosynthesis and storage in the seed oil body may increase the OC of *B. rapa*. This study thus unravels the regulatory mechanisms underlying the variation of SZ, SC, and OC in *B. rapa* seeds, and may facilitate genetic engineering efforts to breed *Brassica* crops with improved yield and OC.

## Materials and methods

### Plant materials

Seeds of two *B. rapa* accessions, SWUK3 (small black seeds with lower OC) and SWUK4 (large yellow seeds with higher OC), were obtained from the Chongqing Rapeseed Engineering Research Center. All seeds were sown at the beginning of October, 2018, and transferred into the field at Southwest University, Beibei, Chongqing, China (29° 45′ N, 106° 22′ E, 238.57 m), 1 month later. Each accession was planted in a plot of five rows, 10 plants per row, with 20 cm between plants within each row, and 30 cm between rows. Based on the field trail, the duration time from flowering to final maturation of *B. rapa* accessions were similar, about 53 and 56 days for SWUK3 and SWUK4, respectively.

### Phenotype measurements

Seeds were collected at seven seed developmental stages: 7, 14, 21, 28, 35, 42, and 49 DAP. Fifty seeds were harvested from the siliques on the main inflorescences at each stage to measure SZ. Based on the Biologische Bundesanstalt, Bundessortenamt, and CHemical (BBCH) industry scale [61], six representative plants from the middle of each plot were harvested at growth stage 99 (harvested product) for measurement of SZ, TSW, and SL. The mature seed OC and seed coat transparency were assayed using NIRS (DS2500) with previously established models for *Brassica* crops [62]. The FA composition, specifically the compositions of palmitic acid, stearic acid, oleic acid, linoleic acid, and linolenic acid, was analyzed by gas–liquid chromatography on a Perkin Elmer Gas Chromatograph Model GC-2010 (Shimadzu, Kyoto, Japan) equipped with a fused silica capillary column DB-WAX (30 m × 0.25 mm id, 0.25 μm film thickness, J&W, Folsom, CA, USA), as reported previously [63]. The OC in seeds harvested from seven developmental stages was assayed by GC–MS, as previously described [64]. For each trait, at least five independent biological replicates were measured.

### Transcriptome sequencing and identification of DEGs

To compare the transcriptomic variations between the two *B. rapa* accessions, a total of 42 seed samples (2 accessions × 7 stages × 3 biological replicates) were collected. Total RNAs of abovementioned samples were isolated using an RNAprep Pure Plant Kit (Tiangen, Beijing, China), and sent to Novogene Corporation (Beijing, China) for library construction and transcriptome sequencing on an Illumina HiSeq 2500 platform. The raw sequencing data were deposited in the BIG Data Center (BIGD) under BioProject accession number PRJCA002339.

Low-quality reads, connectors, and barcode sequences were eliminated using Trimmomatic-0.39 [65] Then, the clean data were aligned to the *B. rapa* reference genome, v3.0 (http://brassicadb.org/brad/), using STAR-2.5.3 [66]. Gene expression levels were quantified as count number and FPKM using the programs featurecounts and cuffquant, respectively [67]. The correlation relationships among all the samples were examined by principal component analysis (PCA), and the correlation coefficients were determined through the R package ggfortify and SPSS15.0 [68]. DEGs were identified using the R package DEseq 2, based on the criteria of false discovery rate (FDR, Benjamini–Hochberg multiple test correction) < 0.01 and absolute fold change > 2 [69].

### Identification of transcription factors, and genes associated with SC and acyl-lipid metabolism

To identify and classify transcription factors (TFs), transcription repressors (TRs), and kinases, all the protein sequences of *B. rapa* were analyzed using the online program iTAK v1.6, (http://itak.feilab.net) [70].

Since the SC and OC differed significantly between the two *B. rapa* accessions, we obtained all of the genes involved in the flavonoid pathway that play critical roles in SC formation, as well as those involved in acyl-lipid metabolism, in Arabidopsis from the Arabidopsis Acyl-Lipid Metabolism database (ARALIP) (http://aralip.plantbiology.msu.edu) [71]. Subsequently, a reciprocal BLASTP with an *E*-value cut-off of 1E−5 was used to identify the homologous relationships between the Arabidopsis and *B. rapa* genes [72], and then the protein sequences were analyzed with Pfam Scan (https://www.ebi.ac.uk/Tools/pfa/pfamscan/) to further confirm the existence of corresponding functional domains.

### *K*-means clustering of DEGs

*K*-means clustering is an effective approach to identify gene expression patterns for transcriptome data. To establish the expression profiles of DEGs with potential biological function in regulating our target traits in *B. rapa*, cluster analysis was performed by the *K*-means method with Pearson’s correlation distance using the cluster package in R. Optimal number of clusters for *K*-means were estimated by the gap statistic that computed using the clusGap function in R package factoextra [73]. *K*-means clustering was then performed with the optimal cluster number 12 for both two *B. rapa* accessions. Heatmaps were created using the expression values with log_2_(FPKM + 1) and *Z*-score normalization and visualized using the pheatmap package in R.

### Weighted gene co-expression network analysis (WGCNA)

To detect co-expression modules and key regulatory genes associated with target seed traits in *B. rapa*, we generated co-expression networks using the WGCNA package in R as previously described [74]. Briefly, only expressed genes with average FPMK values higher than 1 in any seeds were retained and were then log_2_(FPKM + 1) transformed before further processing. The soft thresholding power was determined using pickSoftThreshold function based on the scale-free topology model fit (*R*^2^) > 0.9. Then, the automatic blockwiseModules network construction approach was applied to obtain the highly correlated modules, with the following parameters: power, 18; TOM-type, unsigned; miniModuleSize, 50; maxBlockSize, 35,000; mergeCutHeight, 0.25. The Plant Transcriptional Regulatory Map (PlantRegMap, http://plantregmap.gao-lab.org) was used to analyze regulatory relationships between TFs and their co-expressed genes in the same network [75]. The co-expression and transcriptional regulatory networks were displayed using Cytoscape v3.5.1 [76].

### GO and KEGG enrichment analyses

All the *B. rapa* genes were annotated with BLASTP against the Arabidopsis proteome (TAIR10) with an *E*-value cut-off of 1E−5 (Altschul et al. [72]). GO enrichment analysis was performed using the BiNGO plug-in in Cytoscape v3.5.1 [76]. Significantly overrepresented GO terms were identified with the threshold of FDR < 0.05. The online OmicShare tool (https://www.omicshare.com/tools), a free online platform, was used to perform KEGG pathway enrichment analysis. Bubble plots for GO terms and KEGG pathways were generated using the R package ggplot2 [77].

### qRT-PCR validation

To confirm the accuracy of transcriptomic sequencing and identification of DEGs, the cDNA was synthesized from 1 μg of total RNA that used for transcriptomic sequencing using a PrimeScript RT Master Mix Kit (TaKaRa, Dalian, China). A total of 15 DEGs (3, 4, and 8 genes involved in flavonoid pathway, acyl-lipid metabolism, and seed development, respectively) were selected for qRT-PCR assays. All gene-specific primers were retrieved from the qPrimerDB database (https://biodb.swu.edu.cn/qprimerdb) [78]. All qRT-PCR assays were carried out following the Minimum Information for Publication of Quantitative Real-Time PCR Experiments (MIQE) guidelines. *Bna.UBC21* and *BnaACT7* were used as internal controls, and relative expression levels were calculated using the 2^−ΔΔCt^ method [25].

## Supplementary information

**Additional file 1: Table S1.** Phenotypic traits of two *B. rapa* accessions. **Table S2.** Overview of transcriptomic data and mapping efficiency. **Table S3.** Classification and overview of DEGs. **Table S4.** Identification of classification of all TF, TR and kinase genes in *B. rapa*. **Table S5.** GO enrichment analysis of DEGs obtained by comparison two accessions at the seven stages. **Table S6.** GO enrichment analysis of DEGs obtained by comparison the adjacent stages in each one accession. **Table S7.** Homologous cell cycle genes in *Arabidopsis* and *B. rapa*. **Table S8.** GO enrichment analysis of color modules in WGCNA. **Table S9.** Expression values of flavonoid pathway genes in two *B. rapa* accessions at seven seed developmental stages. **Table S10.** Expression values of genes involved in fatty acid metabolism in two *B. rapa* accessions at seven seed developmental stages. **Table S11.** Primers used in qRT-PCR.

**Additional file 2: Fig. S1.** PCA and coefficient of association analysis. (a) The 2D PCA analysis of SWUK3, The biological replicates of the same period have the same color; (b) Heatmap of all retained samples correlation coefficient of SWUK3. In the upper right corner is the red circle representing relevance, and the bottom left is the correlation coefficient. The higher the value of correlation coefficient, the redder the color; (c) The 2D PCA of SWUK4; (d) Heatmap of all retained samples correlation coefficient of SWUK4. **Fig. S2.** The process of co-expression network construction. (a) The clustering tree of all 38 samples; (b) The left one displays the relationship between the soft threshold and scale independence with 0.9 red line. The right one displays the relationship between the soft threshold and mean connectivity; (c) The cluster dendrogram; (d) Heatmap of the correlation among the color modules, the higher the correlation, the color more deep. **Fig. S3.** Module-trait relationships and GO enrichment of interested color modules. (a) Heatmap of the correlation between the 15 color modules and 4 traits, with correlation coefficient and *P* value; (b) GO enrichment analysis for genes in the MEgreen; (c) GO enrichment analysis for genes in the MEsalmon; (d) GO enrichment analysis for genes in the MEbrown. Only top twenty GO terms were displayed here. **Fig. S4.** Co-expression network of MEgreen and *Bra.A09GRAS*. **Fig S5.** The WGCNA MEbrown module is significantly associated with OC increase. (a) Heatmap of MEbrown module genes in two *B. rapa* accessions, which was displayed based on log2(FPKM + 1). (b) Primary co-expression network of *Bra.A03GRF5*. (c) Primary co-expression network of *Bra.A09WRI1*. (d) Primary co-expression network of *Bra.A06FUS3*. Square, down arrow, triangle, and disc represent TF, TR, kinase, and other genes, respectively. The edge width represents the weight value between the two nodes: the higher the value of the weight between the nodes, the wider the edge.

## Data Availability

The datasets supporting the conclusions of this article are included within the article and its additional files.

## Abbreviations

AP2: APETALA2
ARALIP: Arabidopsis Acyl-Lipid Metabolism database
BB: BIG BROTHER
BIGD: BIG Data Center
BR: Brassinosteroid
CK: Cytokinin
CKX2: CYTOKININ OXIDASE 2
DAP: Days after pollination
DEG: Differentially expressed gene
DGAT: Diacylglycerol acyltransferase
EOD1: ENHANCER OF DA1
FA: Fatty acid
FAD: FA Desaturase
FAX1: Fatty Acid Export 1
FPKM: Fragments per kilobase per million mapped reads
GC: Gas chromatography
GO: Gene Ontology
GPAT9: Glycerol-3-Phosphate Acyltransferase 9
GRF4: GROWTH-REGULATING FACTORS
IKU: HAIKU
KAS II: 3-Ketoacyl-ACP synthase II
KEGG: Kyoto Encyclopedia of Genes and Genomes
LACS9: Long Chain Acyl-Coa Synthetase 9
LPAAT: 1-Acylglycerol-3-phosphate acyltransferase
LPCAT: Lysophosphatidylcholine acyltransferase
MIQE: Minimum Information for Publication of Quantitative Real-Time PCR Experiments
NIRS: Near-infrared reflection spectroscopy
OC: Oil content
PAP: Phosphatidic acid phosphatase
PCA: Principal component analysis
qRT-PCR: Quantitative real-time PCR
SAD: Stearoylacyl Carrier Protein Desaturase
SC: Seed color
SFAR: Seed Fatty Acid Reducer
SHB1: SHORT HYPOCOTYL UNDER BLUE 1
SL: Silique length
SOD2: SUPPRESSOR OF DA1
SPL13: SQUAMOSA PROMOTER BINDING PROTEIN-LIKE
SZ: Seed size
TAG: Triacylglycerols
TF: transcription factor
TR: Transcription repressor
TSW: Thousand-seed weight
TT: TRANSPARENT TESTA
UBP15: UBIQUITIN-SPECIFIC PROTEASE 15
WGCNA: Weighted gene co-expression network analysis
