# Supplementary material for: Deciphering the transcriptional regulatory networks that control size, color, and oil content in Brassica rapa seeds
Source: Biotechnol Biofuels. 2020 May 18;13:90. doi: 10.1186/s13068-020-01728-6 (PMC7236191; doi:10.1186/s13068-020-01728-6)
Supplement: Supplementary file 1 — Additional file 1: Table S1. Phenotypic traits of two B. rapa accessions. Table S2. Overview of transcriptomic data and mapping efficiency. Table S3. Classification and overview of DEGs. Table S4. Identification of classification of all TF, TR and kinase genes in B. rapa. Table S5. GO enrichment analysis of DEGs obtained by comparison two accessions at the seven stages. Table S6. GO enrichment analysis of DEGs obtained by comparison the adjacent stages in each one accession. Table S7. Homologous cell cycle genes in Arabidopsis and B. rapa. Table S8. GO enrichment analysis of color modules in WGCNA. Table S9. Expression values of flavonoid pathway genes in two B. rapa accessions at seven seed developmental stages. Table S10. Expression values of genes involved in fatty acid metabolism in two B. rapa accessions at seven seed developmental stages. Table S11. Primers used in qRT-PCR. [file 13068_2020_1728_MOESM1_ESM.zip › Table S1.docx]

**Additional Table S1 Phenotypic traits of two *B. rapa* accessions**

| Traits | Stage | Accession number | |
| --- | --- | --- | --- |
|  |  | **SWUK3** | **SWUK4** |
| Seed diameters (mm Mean±SD) | 7 DAP | 0.43±0.06 | 0.53±0.07 |
|  | 14DAP | 0.83±0.08 | 1.00±0.06 |
|  | 21 DAP | 1.07±0.07 | 1.21±0.07 |
|  | 28 DAP | 1.82±0.08 | 2.15±0.05 |
|  | 35 DAP | 1.88±0.05 | 2.78±0.05 |
|  | 42 DAP | 1.91±0.06 | 2.88±0.07 |
|  | 49 DAP | 1.81±0.04 | 2.91±0.04 |
|  | Mature dry seed | 1.23±0.01 | 2.42±0.05 |
| Seed diameters variation (mm Mean±SD) | 7-14DAP | 0.40±0.006 | 0.47±0.007 |
|  | 14-21DAP | 0.24±0.005 | 0.21±0.005 |
|  | 21-28DAP | 0.75±0.004 | 0.94±0.009 |
|  | 28-35DAP | 0.16±0.005 | 0.63±0.002 |
|  | 35-42DAP | 0.06±0.003 | 0.10±0.003 |
|  | 42-49DAP | 0.03±0.002 | 0.03±0.001 |
| Palmitic acid | Mature dry seed | 2.80±0.11 | 2.17±0.08 |
| Stearic acid | Mature dry seed | 1.10±0.05 | 1.54±0.05 |
| Oleic acid | Mature dry seed | 12.78±1.46 | 19.61±0.71 |
| Linoleic acid | Mature dry seed | 15.06±0.25 | 12.01±0.31 |
| Linolenic acid | Mature dry seed | 11.17±0.49 | 6.34±0.39 |
| Seed coat transparency | Mature dry seed | 85.45±4.15 | 157.33±2.30 |
| Oil content (% Mean±SD) | 7DAP | 0 | 0 |
|  | 14DAP | 2.51±0.55 | 2.82±1.34 |
|  | 21 DAP | 6.76±0.71 | 7.12±0.89 |
|  | 28 DAP | 11.87±2.04 | 15.26±1.1 |
|  | 35 DAP | 29.89±1.22 | 34.5±1.55 |
|  | 42 DAP | 38.2±0.95 | 43.61±2.58 |
|  | 49 DAP | 39.13±1.26 | 45.81±1.49 |
|  | Mature dry seed | 38.93±0.18 | 42.15±0.12 |
| Seed oil content increase (% Mean±SD) | 7-14DAP | 2.51±0.33 | 2.82±1.02 |
|  | 14-21DAP | 4.25±0.56 | 4.3±0.77 |
|  | 21-28DAP | 5.11±1.22 | 8.14±1.05 |
|  | 28-35DAP | 18.02±1.49 | 19.24±2.84 |
|  | 35-42DAP | 8.31±1.02 | 9.11±0.8 |
|  | 42-49DAP | 0.93±0.21 | 2.2±0.54 |
