# Supplementary figures and images for: Deciphering the transcriptional regulatory networks that control size, color, and oil content in Brassica rapa seeds
Source: Biotechnol Biofuels. 2020 May 18;13:90. doi: 10.1186/s13068-020-01728-6 (PMC7236191; doi:10.1186/s13068-020-01728-6)

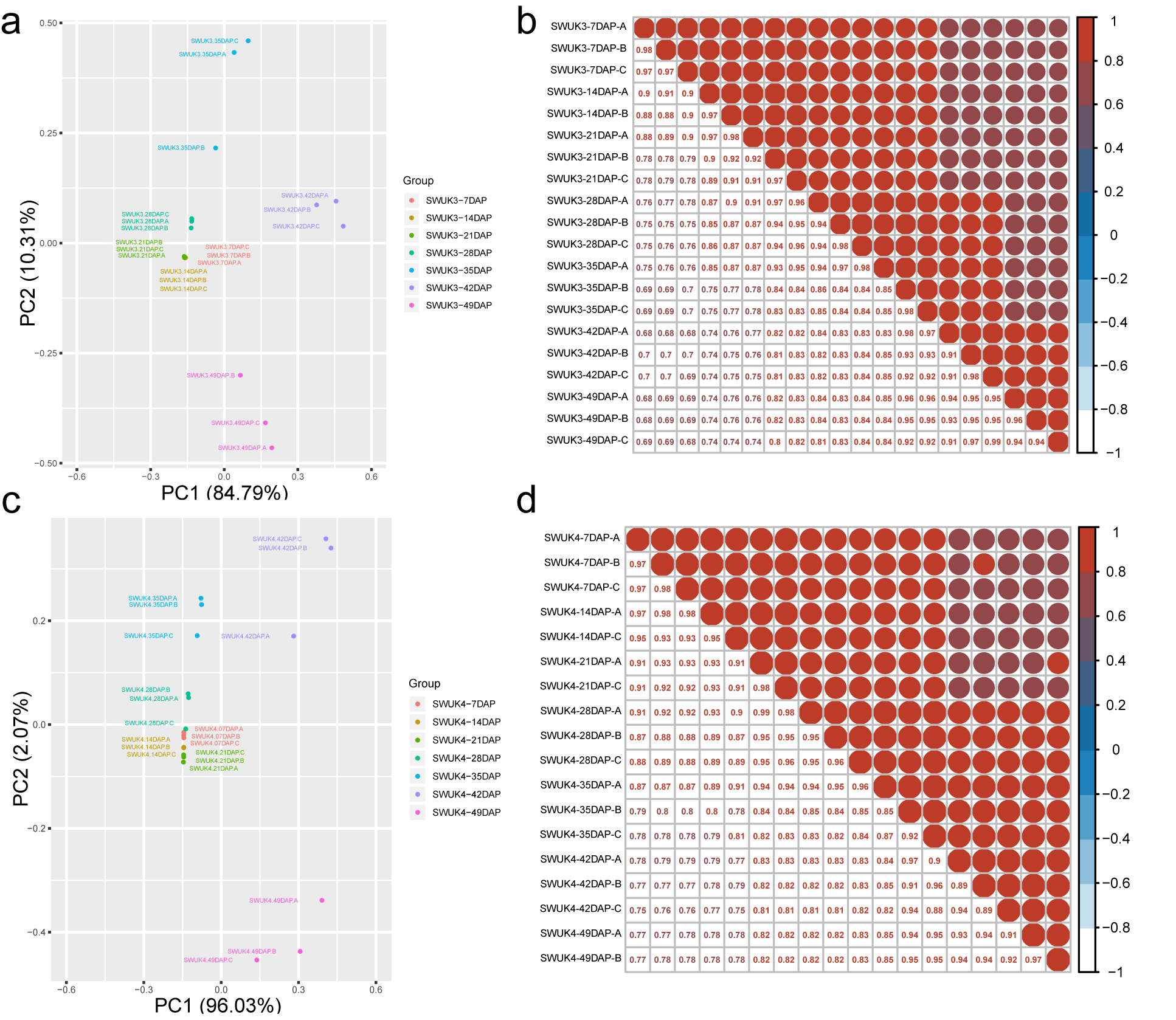

Supplement: Supplementary file 2 — Additional file 2: Fig. S1. PCA and coefficient of association analysis. (a) The 2D PCA analysis of SWUK3, The biological replicates of the same period have the same color; (b) Heatmap of all retained samples correlation coefficient of SWUK3. In the upper right corner is the red circle representing relevance, and the bottom left is the correlation coefficient. The higher the value of correlation coefficient, the redder the color; (c) The 2D PCA of SWUK4; (d) Heatmap of all retained samples correlation coefficient of SWUK4. Fig. S2. The process of co-expression network construction. (a) The clustering tree of all 38 samples; (b) The left one displays the relationship between the soft threshold and scale independence with 0.9 red line. The right one displays the relationship between the soft threshold and mean connectivity; (c) The cluster dendrogram; (d) Heatmap of the correlation among the color modules, the higher the correlation, the color more deep. Fig. S3. Module-trait relationships and GO enrichment of interested color modules. (a) Heatmap of the correlation between the 15 color modules and 4 traits, with correlation coefficient and P value; (b) GO enrichment analysis for genes in the MEgreen; (c) GO enrichment analysis for genes in the MEsalmon; (d) GO enrichment analysis for genes in the MEbrown. Only top twenty GO terms were displayed here. Fig. S4. Co-expression network of MEgreen and Bra.A09GRAS. Fig S5. The WGCNA MEbrown module is significantly associated with OC increase. (a) Heatmap of MEbrown module genes in two B. rapa accessions, which was displayed based on log2(FPKM + 1). (b) Primary co-expression network of Bra.A03GRF5. (c) Primary co-expression network of Bra.A09WRI1. (d) Primary co-expression network of Bra.A06FUS3. Square, down arrow, triangle, and disc represent TF, TR, kinase, and other genes, respectively. The edge width represents the weight value between the two nodes: the higher the value of the weight between the nodes, the wider the ed [file 13068_2020_1728_MOESM2_ESM.zip › Fig. S1.tif]

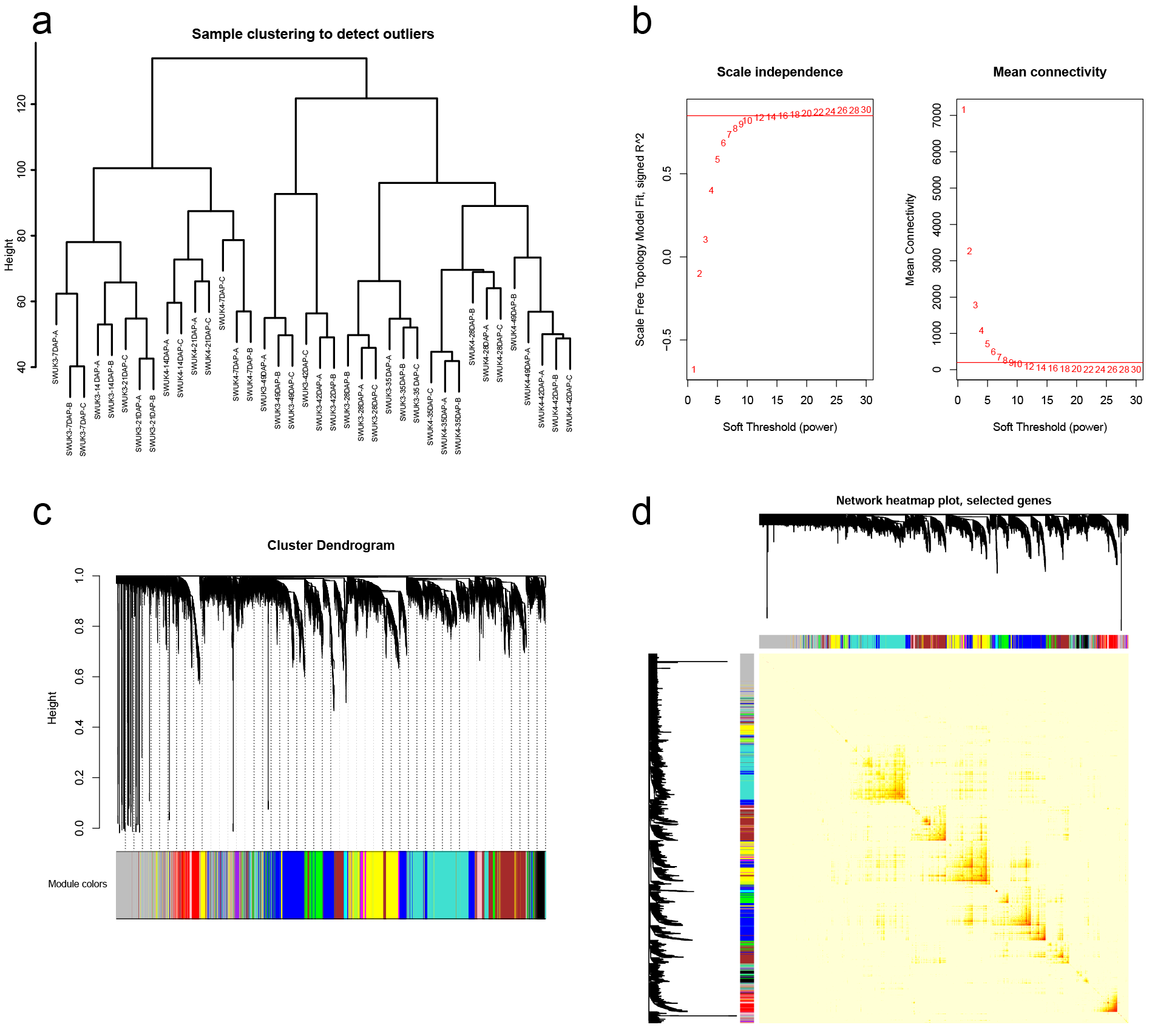

Supplement: Supplementary file 2 — Additional file 2: Fig. S1. PCA and coefficient of association analysis. (a) The 2D PCA analysis of SWUK3, The biological replicates of the same period have the same color; (b) Heatmap of all retained samples correlation coefficient of SWUK3. In the upper right corner is the red circle representing relevance, and the bottom left is the correlation coefficient. The higher the value of correlation coefficient, the redder the color; (c) The 2D PCA of SWUK4; (d) Heatmap of all retained samples correlation coefficient of SWUK4. Fig. S2. The process of co-expression network construction. (a) The clustering tree of all 38 samples; (b) The left one displays the relationship between the soft threshold and scale independence with 0.9 red line. The right one displays the relationship between the soft threshold and mean connectivity; (c) The cluster dendrogram; (d) Heatmap of the correlation among the color modules, the higher the correlation, the color more deep. Fig. S3. Module-trait relationships and GO enrichment of interested color modules. (a) Heatmap of the correlation between the 15 color modules and 4 traits, with correlation coefficient and P value; (b) GO enrichment analysis for genes in the MEgreen; (c) GO enrichment analysis for genes in the MEsalmon; (d) GO enrichment analysis for genes in the MEbrown. Only top twenty GO terms were displayed here. Fig. S4. Co-expression network of MEgreen and Bra.A09GRAS. Fig S5. The WGCNA MEbrown module is significantly associated with OC increase. (a) Heatmap of MEbrown module genes in two B. rapa accessions, which was displayed based on log2(FPKM + 1). (b) Primary co-expression network of Bra.A03GRF5. (c) Primary co-expression network of Bra.A09WRI1. (d) Primary co-expression network of Bra.A06FUS3. Square, down arrow, triangle, and disc represent TF, TR, kinase, and other genes, respectively. The edge width represents the weight value between the two nodes: the higher the value of the weight between the nodes, the wider the ed [file 13068_2020_1728_MOESM2_ESM.zip › Fig. S2.tif]

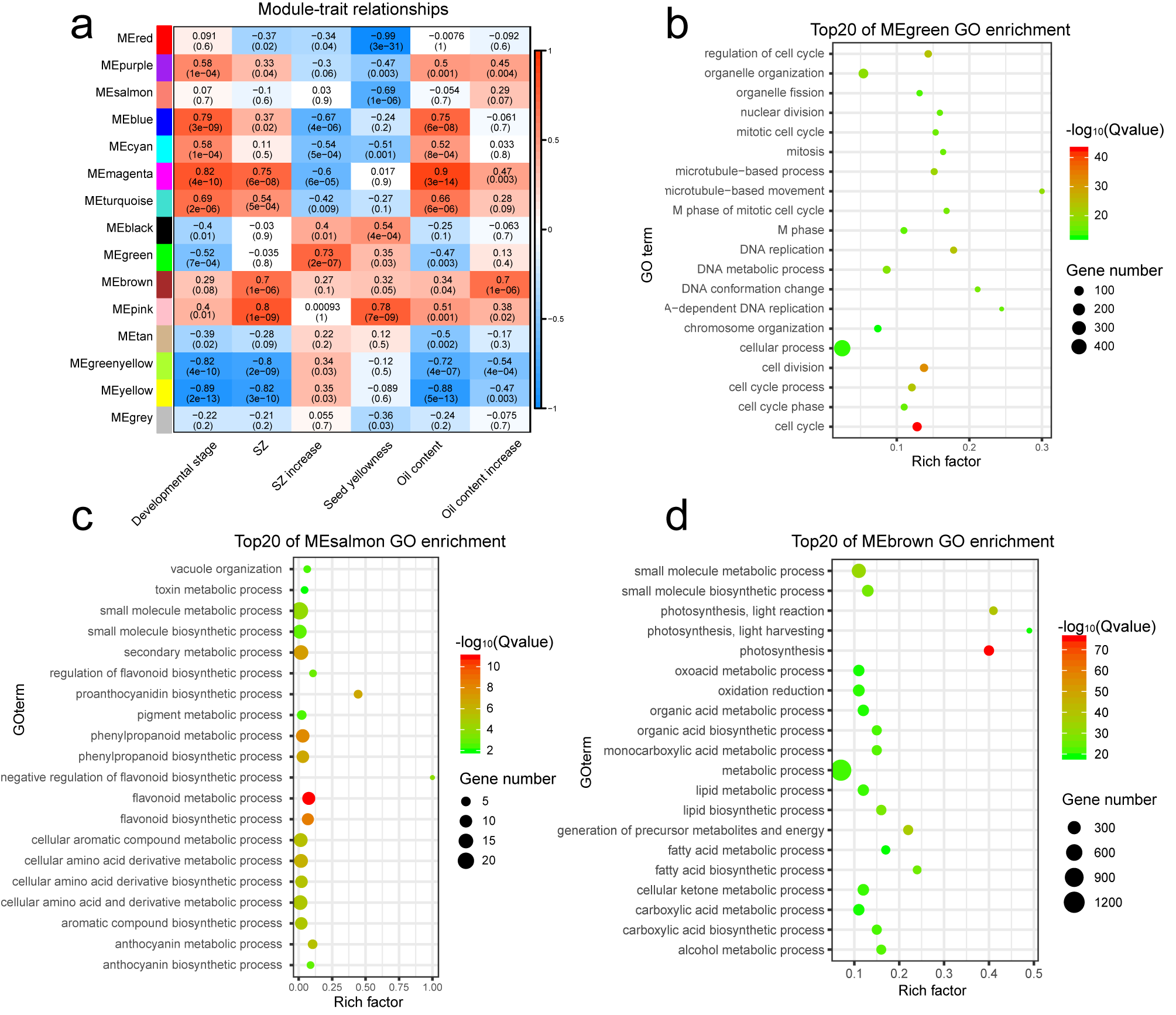

Supplement: Supplementary file 2 — Additional file 2: Fig. S1. PCA and coefficient of association analysis. (a) The 2D PCA analysis of SWUK3, The biological replicates of the same period have the same color; (b) Heatmap of all retained samples correlation coefficient of SWUK3. In the upper right corner is the red circle representing relevance, and the bottom left is the correlation coefficient. The higher the value of correlation coefficient, the redder the color; (c) The 2D PCA of SWUK4; (d) Heatmap of all retained samples correlation coefficient of SWUK4. Fig. S2. The process of co-expression network construction. (a) The clustering tree of all 38 samples; (b) The left one displays the relationship between the soft threshold and scale independence with 0.9 red line. The right one displays the relationship between the soft threshold and mean connectivity; (c) The cluster dendrogram; (d) Heatmap of the correlation among the color modules, the higher the correlation, the color more deep. Fig. S3. Module-trait relationships and GO enrichment of interested color modules. (a) Heatmap of the correlation between the 15 color modules and 4 traits, with correlation coefficient and P value; (b) GO enrichment analysis for genes in the MEgreen; (c) GO enrichment analysis for genes in the MEsalmon; (d) GO enrichment analysis for genes in the MEbrown. Only top twenty GO terms were displayed here. Fig. S4. Co-expression network of MEgreen and Bra.A09GRAS. Fig S5. The WGCNA MEbrown module is significantly associated with OC increase. (a) Heatmap of MEbrown module genes in two B. rapa accessions, which was displayed based on log2(FPKM + 1). (b) Primary co-expression network of Bra.A03GRF5. (c) Primary co-expression network of Bra.A09WRI1. (d) Primary co-expression network of Bra.A06FUS3. Square, down arrow, triangle, and disc represent TF, TR, kinase, and other genes, respectively. The edge width represents the weight value between the two nodes: the higher the value of the weight between the nodes, the wider the ed [file 13068_2020_1728_MOESM2_ESM.zip › Fig. S3.tif]

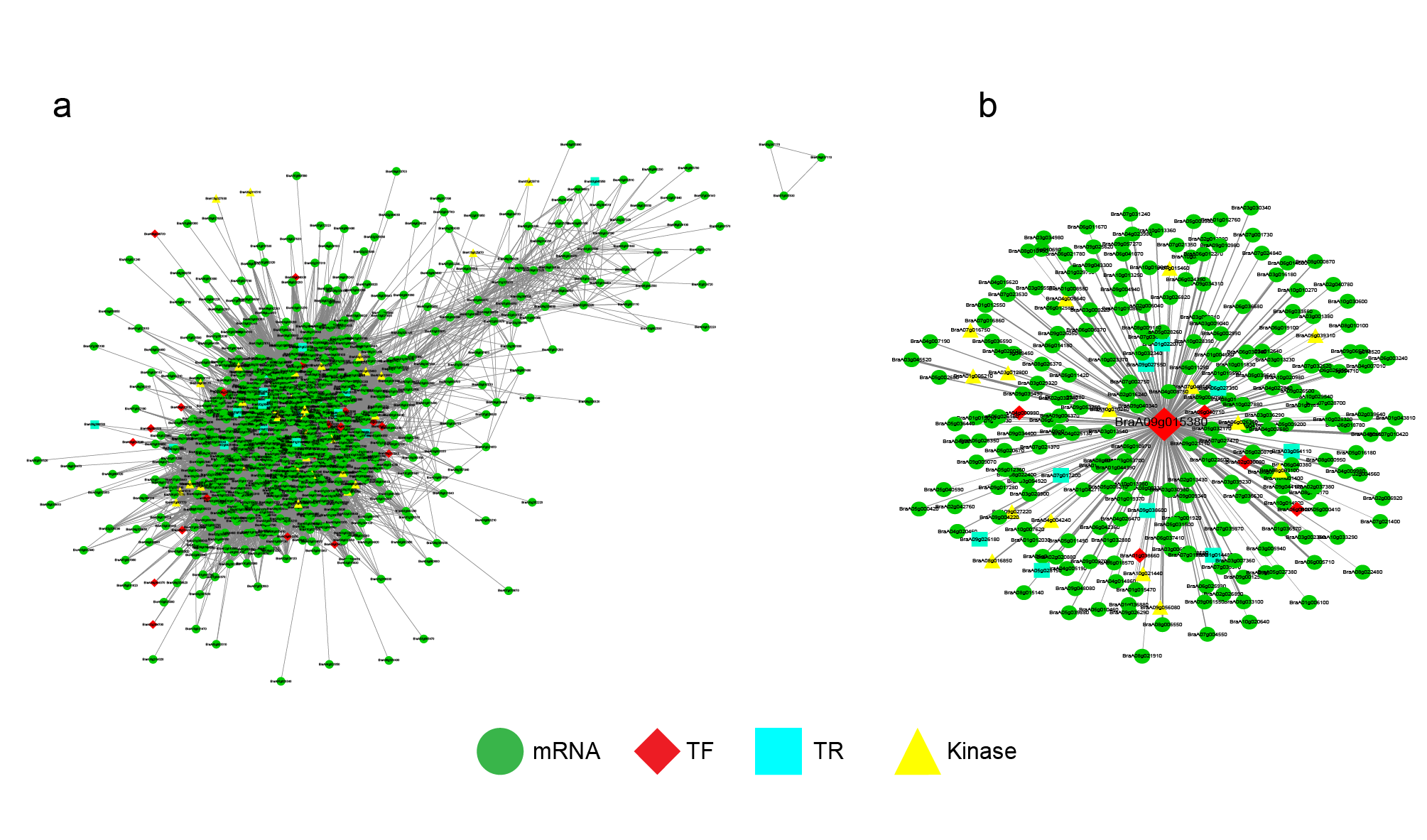

Supplement: Supplementary file 2 — Additional file 2: Fig. S1. PCA and coefficient of association analysis. (a) The 2D PCA analysis of SWUK3, The biological replicates of the same period have the same color; (b) Heatmap of all retained samples correlation coefficient of SWUK3. In the upper right corner is the red circle representing relevance, and the bottom left is the correlation coefficient. The higher the value of correlation coefficient, the redder the color; (c) The 2D PCA of SWUK4; (d) Heatmap of all retained samples correlation coefficient of SWUK4. Fig. S2. The process of co-expression network construction. (a) The clustering tree of all 38 samples; (b) The left one displays the relationship between the soft threshold and scale independence with 0.9 red line. The right one displays the relationship between the soft threshold and mean connectivity; (c) The cluster dendrogram; (d) Heatmap of the correlation among the color modules, the higher the correlation, the color more deep. Fig. S3. Module-trait relationships and GO enrichment of interested color modules. (a) Heatmap of the correlation between the 15 color modules and 4 traits, with correlation coefficient and P value; (b) GO enrichment analysis for genes in the MEgreen; (c) GO enrichment analysis for genes in the MEsalmon; (d) GO enrichment analysis for genes in the MEbrown. Only top twenty GO terms were displayed here. Fig. S4. Co-expression network of MEgreen and Bra.A09GRAS. Fig S5. The WGCNA MEbrown module is significantly associated with OC increase. (a) Heatmap of MEbrown module genes in two B. rapa accessions, which was displayed based on log2(FPKM + 1). (b) Primary co-expression network of Bra.A03GRF5. (c) Primary co-expression network of Bra.A09WRI1. (d) Primary co-expression network of Bra.A06FUS3. Square, down arrow, triangle, and disc represent TF, TR, kinase, and other genes, respectively. The edge width represents the weight value between the two nodes: the higher the value of the weight between the nodes, the wider the ed [file 13068_2020_1728_MOESM2_ESM.zip › Fig. S4.tif]

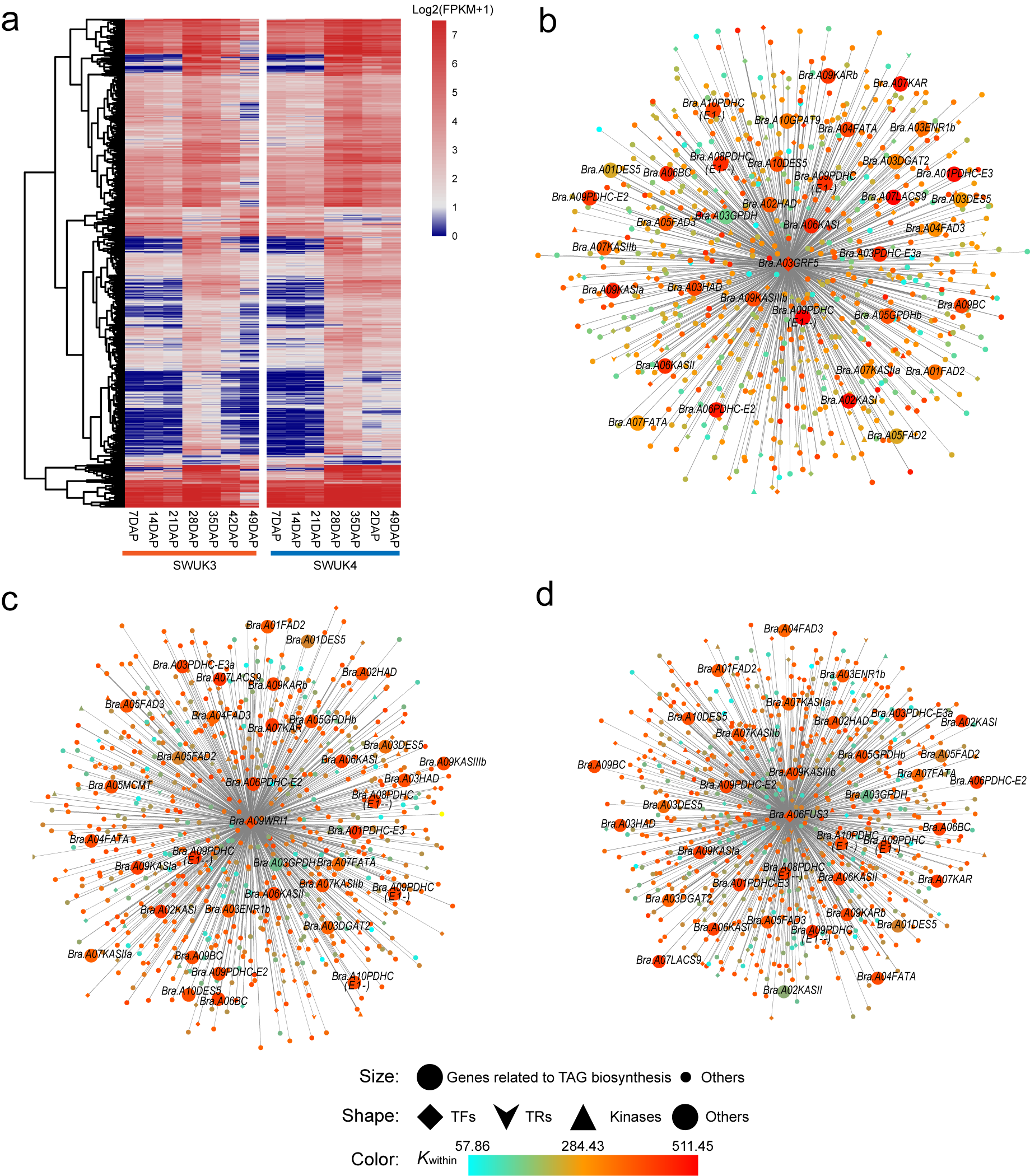

Supplement: Supplementary file 2 — Additional file 2: Fig. S1. PCA and coefficient of association analysis. (a) The 2D PCA analysis of SWUK3, The biological replicates of the same period have the same color; (b) Heatmap of all retained samples correlation coefficient of SWUK3. In the upper right corner is the red circle representing relevance, and the bottom left is the correlation coefficient. The higher the value of correlation coefficient, the redder the color; (c) The 2D PCA of SWUK4; (d) Heatmap of all retained samples correlation coefficient of SWUK4. Fig. S2. The process of co-expression network construction. (a) The clustering tree of all 38 samples; (b) The left one displays the relationship between the soft threshold and scale independence with 0.9 red line. The right one displays the relationship between the soft threshold and mean connectivity; (c) The cluster dendrogram; (d) Heatmap of the correlation among the color modules, the higher the correlation, the color more deep. Fig. S3. Module-trait relationships and GO enrichment of interested color modules. (a) Heatmap of the correlation between the 15 color modules and 4 traits, with correlation coefficient and P value; (b) GO enrichment analysis for genes in the MEgreen; (c) GO enrichment analysis for genes in the MEsalmon; (d) GO enrichment analysis for genes in the MEbrown. Only top twenty GO terms were displayed here. Fig. S4. Co-expression network of MEgreen and Bra.A09GRAS. Fig S5. The WGCNA MEbrown module is significantly associated with OC increase. (a) Heatmap of MEbrown module genes in two B. rapa accessions, which was displayed based on log2(FPKM + 1). (b) Primary co-expression network of Bra.A03GRF5. (c) Primary co-expression network of Bra.A09WRI1. (d) Primary co-expression network of Bra.A06FUS3. Square, down arrow, triangle, and disc represent TF, TR, kinase, and other genes, respectively. The edge width represents the weight value between the two nodes: the higher the value of the weight between the nodes, the wider the ed [file 13068_2020_1728_MOESM2_ESM.zip › Fig.S5.tif]
